# Exploring Novel E3 Ligases and Neosubstrates for Molecular Glue Degraders and Therapeutic Applications in Cancer

**DOI:** 10.32604/or.2026.073660

**Published:** 2026-05-21

**Authors:** Ji Hoon Jang, Joo-Young Kim, Tae-Jin Lee

**Affiliations:** Department of Anatomy, College of Medicine, Yeungnam University, Daegu, Republic of Korea

**Keywords:** Molecular glue degraders, E3 ubiquitin ligases, anticancer agent, E3 ligase substrate

## Abstract

Molecular glue degraders (MGDs) are an emerging class of small molecules that promote selective protein degradation by inducing neomorphic interactions between E3 ubiquitin ligases and non-native substrates, referred to as neosubstrates. Clinically validated examples include thalidomide analogs that recruit cereblon (CRBN) to degrade IKAROS family zinc finger 1/3 in multiple myeloma, and arylsulfonamide-based MGDs that promote the degradation of RNA-binding protein 39 in acute myeloid leukemia and solid tumors. These molecules highlight the therapeutic potential of this modality in oncology. These findings underscore the promise of MGDs for eliminating oncogenic proteins previously considered undruggable and overcoming resistance to conventional inhibitors. Despite these successes, the current MGD landscape relies heavily on a limited set of E3 ligases-mainly CRBN which constrains substrate diversity, tissue selectivity, and durability of clinical response. Expanding the therapeutic utility of MGDs requires the systematic identification of novel ligases and their neosubstrates, accompanied by a deeper understanding of the mechanistic basis of ligase-substrate recognition. Recent technological advances, including chemoproteomics, ubiquitin-remnant profiling, degron mapping, clustered regularly interspaced short palindromic repeats-based functional genomics, and artificial intelligence-driven structural modeling, are advancing the discovery of new ligase-substrate pairs and enabling the rational design of degraders. Parallel progress in next-generation CRBN E3 ligase modulators, noncanonical MGDs, and structure-guided engineering further illustrates the expanding therapeutic versatility of this approach. By integrating multidisciplinary discovery strategies with translational oncology, the field is moving toward the development of next-generation MGDs with enhanced specificity, broader substrate scope, and improved resistance profiles. This study aims to elucidate how these innovations expand the degradable proteome and establish MGDs as a cornerstone of precision cancer therapy, thereby redefining the boundaries of drug discovery and providing customizable degraders tailored to diverse cancer contexts.

## 1 Introduction

**Figure 1:**
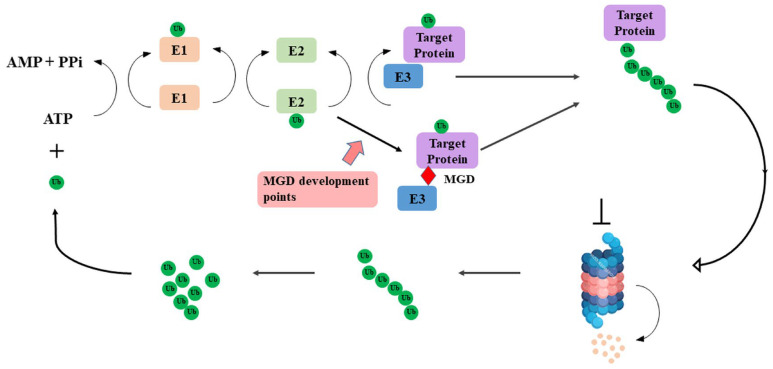
Ubiquitin-proteasome system and Molecular glue degrader (MGD) development point. Schematic representation of the ubiquitin-proteasome pathway, highlighting the sequential action of E1 ubiquitin-activating enzyme, E2 ubiquitin-conjugating enzyme, and E3 ubiquitin ligase in attaching ubiquitin chains to substrate proteins, marking them for degradation by the 26S proteasome. The red arrow highlights the MGD development points, representing sites where small molecules can induce the interaction between E3 ligases and target proteins to promote targeted degradation. The pictures were drawn using PowerPoint. Note: MGD, Molecular glue degrader.

The ubiquitin-proteasome system (UPS) is a fundamental cellular pathway that is central to maintaining homeostasis by selectively degrading damaged, misfolded, or misregulated proteins. This tightly regulated process involves a multistep cascade: An E1 ubiquitin-activating enzyme activates ubiquitin, which is then transferred to an E2 ubiquitin-conjugating enzyme. E3 ubiquitin ligases play a crucial role in determining substrate specificity. Really interesting new gene (RING) and U-box E3 catalyze the direct transfer of ubiquitin from E2 to the substrate, while homologous to the E6AP carboxyl terminus (HECT) and RING-between-RING (RBR) E3 first form a transient E3-ubiquitin thioester intermediate, then transfer ubiquitin to specific lysine residues on the substrate. Once polyubiquitinated, the proteasome, a large multisubunit complex, recognizes the protein, unfolds, and degrades it into short peptides, releasing free ubiquitin for reuse [1,2,3] (Fig. 1).

Beyond its role in protein quality control, the UPS influences various biological processes. It is central in cell cycle control, modulating proteins involved in cell division, and contributes to the immune response through antigen presentation and signaling [4,5,6]. Under cellular stress, the UPS eliminates misfolded proteins, ensuring protein quality control. It also regulates apoptosis (programmed cell death) and DNA repair by modulating relevant proteins. In muscles, it supports homeostasis by controlling protein turnover, while in the nervous system, it prevents the accumulation of toxic protein aggregates, a key feature of neurodegenerative diseases [7,8]. However, UPS dysregulation is associated with numerous pathologies, including cancer-characterized by the inappropriate degradation of tumor suppressors or the stabilization of oncogenic proteins-neurodegenerative disorders such as Alzheimer’s and Parkinson’s diseases, which result from the buildup of misfolded proteins, and autoimmune diseases arising from disrupted immune regulation. The UPS is thus indispensable for cellular integrity, and its proper functioning is essential for health, making it a biological safeguard and a potential therapeutic target.

Targeted protein degradation (TPD) is a transformative therapeutic strategy that co-opts the cell’s natural protein disposal systems, primarily the UPS and occasionally lysosomal pathways-to selectively eliminate disease-relevant proteins [9,10,11,12]. Unlike traditional inhibitors that merely block protein function, TPD aims for the complete removal of target proteins, offering a potentially more effective and durable therapeutic approach [13]. A key advantage of TPD is its ability to address “undruggable” proteins that lack enzymatic activity or well-defined binding pockets.

TPD utilizes several innovative modalities. Proteolysis-targeting chimeras (PROTACs) are bifunctional molecules that simultaneously bind a target protein and an E3 ubiquitin ligase, facilitating the target’s ubiquitination and proteasomal degradation [14]. Molecular glue degraders (MGDs) are smaller, monovalent molecules that induce or stabilize interactions between a target protein and an E3 ligase, also leading to ubiquitin-mediated degradation [12]. In contrast, lysosome-targeting chimeras exploit lysosomal degradation pathways to target both intracellular and extracellular proteins [12]. The therapeutic potential of TPD is currently being explored across oncology, immunology, and neurological diseases [14,15].

MGDs, a distinct class of small molecules, induce TPD by acting as “molecular glues”. They facilitate a neomorphic protein-protein interaction (PPI) between a protein of interest (POI) and an E3 ubiquitin ligase [16,17]. This process brings the two proteins into close proximity, forming a ternary complex that enables the E3 ligase to ubiquitinate the target protein, subsequently leading to its degradation by the proteasome [17,18]. A notable outcome of this mechanism is the recruitment of neosubstrates-substrates not normally recognized by the E3 ligase-which represents a significant aspect of the therapeutic potential of MGD [19,20,21].

MGDs offer key advantages over PROTACs, primarily due to their smaller size and simpler, single-molecule structure [22], which confers more favorable drug-like properties, including improved pharmacological properties and a broader target range. Their lower molecular weight makes them easier to synthesize and leads to better oral absorption. They are also more likely to cross the blood-brain barrier, which is crucial for developing treatments for central nervous system diseases. MGDs can target proteins that were previously considered “undruggable” because they do not require a traditional binding pocket [23]. Instead, they create a new binding surface, allowing for a broader range of potential therapeutic targets.

**Figure 2:**
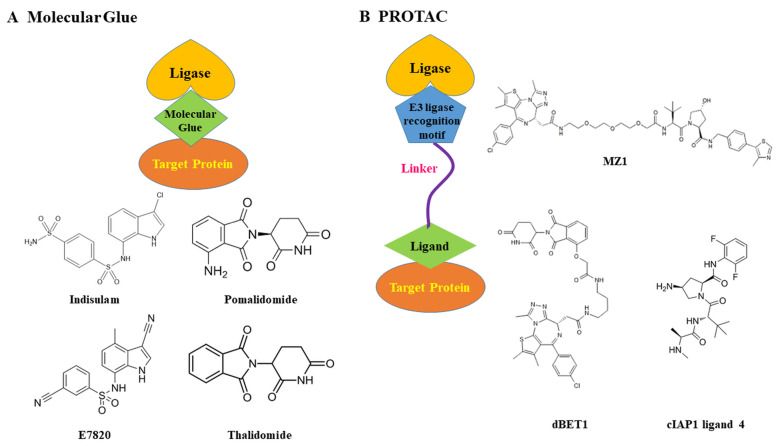
Comparison of MGDs proteolysis-targeting chimeras (PROTACs). (**A**) Schematic diagram of the structure of molecular glue. Molecular glues are small molecules that induce or stabilize interactions between an E3 ligase and a target protein, leading to ubiquitination and degradation. Indisulam, pomalidomide, E7820, and Thalidomide are shown as an example of a clinically approved molecular glue. (**B**) Schematic diagram of the structure of PROTAC. PROTACs are bifunctional molecules composed of an E3 ligase ligand (warhead), a target protein ligand, and a linker that bridges the two, thereby promoting ubiquitination and degradation of the target. MZ-1, dBET1, and cIAP1 ligand 4 are shown as representative PROTACs. The pictures were drawn using PowerPoint. Note: PROTAC, proteolysis-targeting chimera; BET, bromodomain and extra-terminal domain; cIAP1, cellular inhibitor of apoptosis protein 1.

The MGD concept was clinically validated by thalidomide and its derivatives, including lenalidomide and pomalidomide. These FDA-approved drugs, also known as immunomodulatory drugs (IMiDs), bind to the E3 ligase cereblon (CRBN), inducing the degradation of specific proteins. This mechanism is highly effective in treating diseases like multiple myeloma and myelodysplastic syndromes (MDS) [24]. The discovery that these drugs act as MGDs and target CRBN has been a breakthrough, offering a clear understanding of their mechanism and inspiring the development of new TPD therapies [20,21] (Fig. 2).

Thalidomide and its derivatives, including lenalidomide and pomalidomide (known as IMiDs), have achieved significant clinical success by acting as MGDs. These drugs treat multiple myeloma and MDS by binding to the CRBN protein, which is part of an E3 ubiquitin ligase complex [24]. This binding event induces the degradation of specific proteins, such as Ikaros and Aiolos, in multiple myeloma, and casein kinase 1 α (CK1 α) in MDS, ultimately resulting in anticancer effects [25,26]. For example, lenalidomide specifically treats a type of MDS, improving blood counts and reducing the need for transfusions [27]. The discovery of CRBN as the target of thalidomide was a breakthrough that clarified how these drugs work and opened the door for other targeted therapies, such as PROTACs that utilize the CRBN pathway [19].

A key limitation of TPD is its heavy reliance on a few well-characterized E3 ubiquitin ligases, primarily CRBN despite the human genome encoding over 600 E3 ligases [28,29,30]. The scarcity of ligands for other E3 ligases is a major barrier to developing new therapies. The discovery of new MGDs remains challenging, as it is still heavily dependent on empirical screening, often focusing on proteins with specific motifs, such as a β-hairpin that can be recognized by CRBN [31,32].

A critical challenge for MGDs and PROTACs lies in achieving high selectivity. This involves ensuring that the degrader targets only the intended target protein without inducing unwanted off-target effects, particularly since E3 ligases naturally interact with a multitude of proteins [33,34]. To overcome these limitations, current research focuses on identifying novel small-molecule ligands for E3 ligases beyond CRBN. Innovative methodologies, such as the combinatorial mapping of E3 ubiquitin ligases to their target substrates (COMET) and advanced computational modeling, are being utilized to systematically map E3 ligase-substrate relationships and improve the specificity of TPD [28,35,36]. 

This review highlights the urgent need for innovative approaches to identify novel E3 ligases and their cognate neosubstrates, thereby unlocking the full therapeutic potential of MGDs.

## 2 Landscape of E3 Ligases and Their Role in MGDs

**Figure 3:**
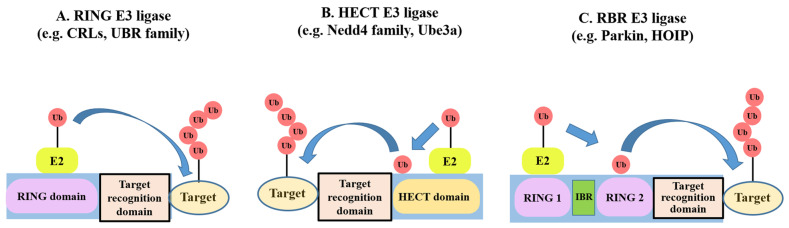
Three major classes of E3 ubiquitin ligases. (**A**) RING E3 ligases (e.g., CRLs, UBR family). RING E3 ligases act as scaffolds, recruiting both the E2-ubiquitin conjugate and the substrate, and directly transferring ubiquitin from E2 to the target protein. CRL achieves specificity via interchangeable substrate receptor proteins (such as VHL). This mechanism is distinct from HECT (**B**) and RBR (**C**) ligases, which generally rely on substrate-recognition domains within a single polypeptide. (**B**) HECT E3 ligases (e.g., Nedd4 family, Ube3a). HECT E3 ligases form a transient thioester intermediate with ubiquitin on their catalytic cysteine within the HECT domain before transferring ubiquitin to the substrate. (**C**) RBR E3 ligases (e.g., Parkin, HOIP). RBR E3 ligases utilize a hybrid mechanism combining RING-mediated E2 recruitment and HECT-like ubiquitin transfer, with ubiquitin first received on a catalytic cysteine and then transferred to the substrate. The pictures were drawn using PowerPoint. Note: RING, Really interesting new gene; CRLs, Cullin-RING Ligases; UBR, ubiquitin-protein ligase E3 component n-recognin: VHL, Von Hippel-Lindau; HECT, homologous to E6-AP carboxyl terminus; RBR, RING-in-between-RING; Nedd4, neural precursor cell expressed developmentally down-regulated protein 4; Ube3a, Ubiquitin-protein ligase E3A; HOIP, HOIL-1-interacting protein.

E3 ubiquitin ligases are the final and most specific component of the three-enzyme cascade (E1-E2-E3) responsible for transferring ubiquitin to specific substrate proteins [37]. This specificity is crucial for regulating vital cellular processes, such as cell cycle progression, DNA repair, and immune responses. E3 ubiquitin ligases are divided into three main families based on their ubiquitin transfer mechanisms: RING, HECT, and RBR [37,38]. E3 ubiquitin ligases are mechanistically categorized based on how they transfer ubiquitin (Ub) from the E2 enzyme to the substrate: RING E3s, such as the CRL family, directly bridge the transfer, while HECT and RBR E3s form a ubiquitin-thioester intermediate before transfer [See Fig. 3]. The Cullin-RING Ligase (CRL) family, which includes VHL, is the primary class of E3 ligases that achieves substrate specificity through interchangeable substrate receptor proteins. In contrast, HECT and RBR ligases employ distinct mechanisms, often relying on integrated recognition domains within a single polypeptide chain [39] (Fig. 3). 

E3 ligase specificity is governed by structural recognition and biological context. For instance, the extensive CRL family utilizes substrate receptors to identify specific degrons on target proteins [40]. To systematically map these interactions, the COMET assay has been developed. When integrated with computational tools like AlphaFold-Multimer, COMET can predict and validate the precise amino acid interfaces that enable highly specific binding between an E3 ligase and its substrate [35]. Beyond structural binding, specificity is further refined through tissue-specific expression. An E3 ligase may be active only within certain tissues-a characteristic that can be strategically exploited in drug design to minimize off-target effects. In this regard, the ELiAH database provides a comprehensive atlas of E3 ligase expression, aiding the selection of tissue-specific E3s for TPD [41]. Additionally, E3 ligase activity is tightly regulated by internal mechanisms such as auto-ubiquitination, which serves to fine-tune its functional output [40].

The inherent ability of E3 ligases to recognize and bind specific substrates makes them a cornerstone of TPD strategies, including MGDs and PROTACs. However, despite the human genome encoding over 600 E3 ligases, current therapeutic strategies rely on only a very small fraction, most notably CRBN [16,42]. These well-characterized ligases represent less than 2% of the total E3 ligase family. The vast majority of E3 ligases remain a “blind spot” in drug discovery, as their unique binding pockets and PPI interfaces are poorly understood [16,43]. This knowledge gap underscores the urgent need for a more comprehensive investigation into their unique characteristics to expand the “PROTACtable universe” of E3 ligases.

Innovative approaches are emerging to address this challenge. For instance, engineered ubiquitin variants can be designed as highly specific modulators to target unique binding sites on E3s [42]. By systematically characterizing E3 ligases based on their ligandability and structural availability, we can unlock the full therapeutic potential of MGDs and other TPD modalities.

### 2.1 RING E3 Ligases

RING E3 ligases constitute the largest family of E3 ligases, characterized by the presence of a RING finger domain. This domain facilitates the direct transfer of ubiquitin from the E2 enzyme to the substrate protein by bringing the E2-ubiquitin conjugate near the target. RING-type E3 ligases do not form a covalent intermediate with ubiquitin themselves; instead, they act as scaffolds that promote the transfer reaction. They are crucial regulators of the stability and activity of numerous interaction substrates [44].

**Figure 4:**
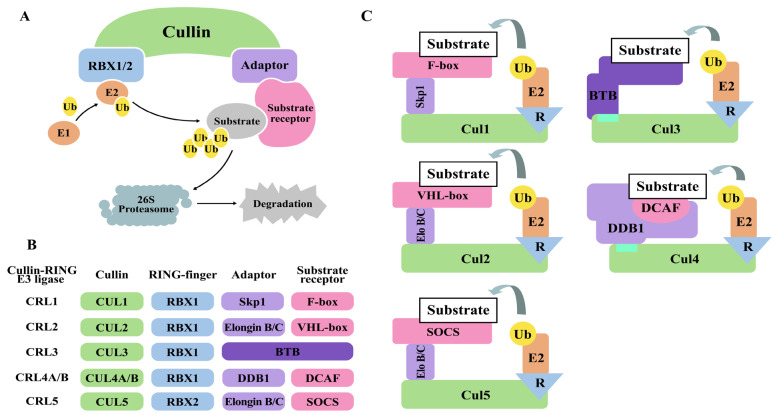
Schematic Diagram of the Structure and Mechanism of Action of the Cullin-RING E3 Ligase (CRL). (**A**) CRLs-mediated Ubiquitination and Substrate Degradation Process. Ubiquitin (Ub) is activated by the E1 enzyme and is subsequently transferred to the E2 enzyme. The Cullin-RING E3 Ligase (CRL) complex functions to attach the Ub from the E2 to a specific substrate (ubiquitination). Cullin (green) acts as the scaffold, and RBX1/2 (blue) is the RING-finger protein that recruits the E2. The substrate receptor (pink/magenta) recognizes and binds to a specific substrate. The Adaptor (light purple) protein links the Cullin to the substrate receptor. The ubiquitinated substrate moves to the 26S Proteasome for degradation. (**B**) Module constructions of different CRLs. CRLs are composed of a Cullin (scaffold, green), a RING-finger protein (RBX1/2), an Adaptor protein (light purple), and a substrate receptor (pink), and are classified according to the combination of these components. The BTB domain protein (dark purple) acts as both an adaptor and substrate receptor. (**C**) Architecture of the CRL complex. CUL1 recruits the adaptor Skp1, which binds to F-box proteins (receptor). CUL2 and CUL5 recruit the adaptor Elongin B/C. CUL2-based CRLs use VHL-box proteins as receptors, while CUL5-based CRLs use SOCS proteins as receptors. CUL3 uses a BTB domain protein (Deep purple) both adaptor and substrate receptor. CUL4A and CUL4B use DDB1 as the adaptor, which recruits DCAF proteins (receptor). The Cullins form a complex with RBX1/2 (R, blue triangle) to constitute the catalytic core of the CRL, and the E2 enzyme charged with Ub is recruited by RBX1/2. The pictures were drawn using Illustrator. Note: Ub, Ubiquitin; RBX, RING-box protein 1/2; BTB, bric-a-brac, tramtrack and broad complex; SOCS, suppressors of cytokine signaling; DCAF, DDB1 and CUL4-associated factor.

This broad family encompasses single-subunit RING E3 ligases, including Mouse double minute 2 (MDM2), Casitas B-lineage lymphoma (CBL), and Tumor necrosis factor receptor-associated factor (TRAF) 6. However, the most significant subclassification of RING E3 ligases is the multisubunit CRLs. These complexes use a Cullin protein as a scaffold to assemble various proteins. Within the CRLs, several key types have been identified [39,45]. The Skp1-Cullin1-F-box (SCF) protein complex is a prime example; it comprises Skp1, Cullin1, Rbx1, and an F-box protein (e.g., β-TrCP and FBXW7) that provides substrate specificity. CRL2 ligases contain Cullin2, famously found in the VHL complex. CRL3 ligases utilize BTB domain proteins as both adaptor and substrate receptor. CRL4 ligases are exemplified by the DDB1-CUL4 complex, where the DDB1- and CUL4-associated factor (DCAF) family members, including CRBN, act as substrate-recognition subunits [45]. Another critical multisubunit RING E3 ligase is the anaphase-promoting complex/cyclosome, essential for cell cycle regulation. The broad substrate specificity of RING E3 ligases and their involvement in diverse cellular pathways make them significant targets for therapeutic intervention (Fig. 4). 

### 2.2 HECT E3 Ligases

HECT E3 ligases operate through a distinct two-step mechanism. Unlike RING ligases, HECT ligases directly accept ubiquitin from E2, forming a transient thioester bond with the ubiquitin molecule via a conserved cysteine residue in their HECT domain. Subsequently, ubiquitin is transferred from the HECT ligase to the lysine residue of the target substrate [46,47]. This unique catalytic mechanism enables more precise control over substrate ubiquitination. Approximately 28 HECT E3 ligases are found in humans, including E6AP (UBE3A), Neural precursor cell expressed developmentally down-regulated protein 4 (NEDD4), ITCH, and SMAD specific E3 ubiquitin protein ligase 1/2 (SMURF1/2). Prominent examples include members of the Nedd4 family, which are involved in regulating membrane protein trafficking and degradation, and Ube3a (E6-AP), which is implicated in Angelman syndrome and the human papillomavirus-mediated degradation of p53 [46].

### 2.3 RBR E3 Ligases

RBR E3 ligases represent a hybrid class that integrates the functional features of both RING and HECT ligases. This family was relatively recently characterized, with approximately 14 RBR E3 ligases identified to date. Structurally, RBR ligases are defined by two RING domains separated by an in-between-RING (IBR) domain. Functionally, they recruit an E2 ubiquitin-conjugating enzyme via their first RING domain (RING1), a mechanism analogous to that of RING ligases. However, they subsequently form a thioester intermediate with ubiquitin on a conserved cysteine residue within their second RING domain (RING2)-akin to the catalytic process of HECT ligases-before finally transferring the ubiquitin to the target protein. This unique two-step mechanism provides an additional layer of regulatory complexity to the ubiquitination process [48,49]. Key examples include Parkin, a critical ligase involved in mitophagy and Parkinson’s disease, and HOIL-1-interacting protein (HOIP), a component of the linear ubiquitin chain assembly complex with a central role in immune signaling and inflammation [48].

### 2.4 Known or Candidate E3 Ligases from MGD Discovery

A key advantage of TPD lies in its exploitation of the inherent flexibility of individual E3 ubiquitin ligases to recognize diverse substrates. This flexibility provides substantial opportunities for developing degraders that selectively target oncoproteins with precision across specific tissues, tumors, and subcellular locations [41]. Ongoing research in induced protein degradation is actively enhancing our understanding of these underlying mechanisms and identifying novel E3 ubiquitin ligase targets, thereby driving the development of more effective and selective therapeutic agents [41].

Despite the vast potential of E3 ubiquitin ligases in the drug discovery landscape, only a small fraction has been effectively harnessed by small-molecule degraders, including MGDs; currently, this remains predominantly limited to CRBN. This constraint underscores a critical need for the continued and expanded exploration of E3 ubiquitin ligase biology to fully establish induced protein degradation as a highly specific and efficient therapeutic strategy [50,51,52].

Significant efforts are underway to address this limitation, including the use of various natural and synthetic small molecules to modulate the activity and substrate recognition of diverse ligases [51,53]. While CRBN has historically been the primary E3 ligase exploited for MGD development, the field is rapidly expanding to include a broader array of E3 ligases [50,51,54]. This diversification is essential to unlocking the full therapeutic potential of MGDs, enabling the precise targeting of a wider range of “undruggable” target proteins and expanding their application across diverse disease contexts [54].

#### 2.4.1 DCAF Family Ligases and Related Adaptors

The DCAF family is a significant group of E3 ligase-substrate receptors that is gaining increased attention [19,55]. CRLs are multisubunit E3 ubiquitin ligases that share a core structure (comprising a Cullin and RING protein) but utilize interchangeable modules for substrate recognition. These complexes are fundamentally organized around two key components: adaptors and substrate receptors. Adaptors are intermediate linker proteins that bridge the Cullin scaffold to the substrate receptor. (e.g., DDB1 in CRL4 complexes, SKP1 in CRL1 complexes). Substrate receptors are subunits that directly recognize and bind the target substrate protein (e.g., DCAF family proteins in CRL4 complexes). The CRL architecture varies significantly, a point best illustrated by comparing CRL4 and CRL3 complexes: CRL4 uses a three-component assembly (CUL4-Adaptor DDB1-Receptor DCAF), while CRL3 uses a simpler two-component assembly (CUL3-Receptor BTB protein), where the BTB receptor binds CUL3 directly, omitting the adaptor protein. This structural contrast highlights the modularity and distinct substrate recruitment mechanisms across the CRL superfamily. BTB domain-containing proteins and DCAFs represent major subfamilies of E3 ligase-substrate receptors. These receptors often feature druggable β-propeller domains, particularly from the WD40 and Kelch families [56,57]. 

WD40 domains are a common structural motif found in many substrate receptors, constituting a major DCAF subclass. They typically form a beta-propeller structure, which is responsible for substrate binding. This WD40 domain is an accurate description of a WD40-repeat-containing beta-propeller fold that directly engages the substrate. The WD40 structural motif is characterized by an approximately 40 amino acid sequence, often ending with the eponymous Tryptophan (W) and Aspartic acid (D) residues [58]. These WD40 repeats assemble into a circular WDR (WD-repeat domain) β-propeller structure, creating a central binding cavity. E3 ligases containing a WD40 domain use this β-propeller surface to recruit specific substrates. In the case of DCAFs, they link the substrate to the larger ubiquitination machinery via the DDB1 adaptor protein of the Cullin 4 (CUL4)-based ligase complex. DCAFs constitute a large group of approximately 60 human proteins, with 52 members comprising a WD40 domain [59]. Notably, while CRBN is a well-studied DCAF, it represents a structural exception as it does not contain a WD40 repeat domain. Instead, it utilizes an alpha-helical bundle domain for substrate recognition and binding [60].

DCAF15: DDB1 and CUL4 Associated Factor (DCAF)15 is a vital substrate-recognition component of the DCX E3 ubiquitin ligase complex. As an adaptor protein within the CRL4 E3 ligase complex, DCAF15 is responsible for recruiting specific target proteins to the core machinery (CUL4A/B, DDB1, and DDA1) for ubiquitination and subsequent degradation. This ligase is notable for its involvement in the degradation of splicing factors, such as RNA-binding protein 39 (RBM39)/RBM23, a process exemplified by the MGD indisulam [61,62]. DCAF15’s capacity to be co-opted by these MGDs to target otherwise undruggable RNA-binding proteins highlights its significant therapeutic promise.

DCAF16: DCAF16 is a substrate-recognition component of the CRL4 E3 ubiquitin ligase complex, much like DCAF15. It is central in the cell’s protein degradation system, bringing target proteins to the CUL4-DDB1 complex for ubiquitination and eventual breakdown. What makes DCAF16 particularly noteworthy is its ability to be targeted by covalent MGDs. These MGDs form strong, covalent bonds with specific cysteine residues on DCAF16 (e.g., Cys58, Cys177–179). As a relatively new and exciting target, DCAF16, along with other DCAF family members, is being actively explored for its potential to recruit MGDs to novel substrates, thus expanding the range of proteins that can be targeted for degradation in drug development [63].

CRBN: CRBN is a substrate receptor for the CRL4 complex. It can be exploited by small molecules to facilitate the recruitment and ubiquitination of non-natural CRL4 substrates, resulting in MGD-induced protein degradation [64]. This phenomenon has emerged as an innovative therapeutic approach, standing in stark contrast to traditional small-molecule drugs. CRBN is a crucial substrate receptor within the CRL4 complex, comprised of DDB1, CUL4, and RBX1. This E3 ligase complex is particularly significant in MGD development and is widely used in this context, among >600 known E3 ligases [29,64].

#### 2.4.2 Ligases with Diverse Cellular and Disease-Related Functions

Beyond the DCAF family, several E3 ligases with diverse cellular roles are being investigated MGDs development. These ligases offer distinct substrate specificities and unique therapeutic opportunities across various disease areas [54,64]. 

A novel small-molecule MGD candidate has been identified to bind within the HIF1α-binding pocket of VHL, inducing a neomorphic interaction with cysteine dioxygenase 1 (CDO1)-a protein not normally recognized by the VHL ligase [65]. This interaction promotes the recruitment of CDO1 into the VHL-Cullin-RING E3 ligase complex, leading to its selective ubiquitination and degradation. The specific surface region of CDO1 responsible for VHL recruitment was elucidated through a combination of mutagenesis, protein-protein docking, and molecular dynamics simulations [65]. Furthermore, the X-ray crystal structure of the ternary complex (VHL-CDO1-degrader) validated these findings, revealing the atomic-level interactions that underpin this molecular glue mechanism.

MDM2 can be targeted by MGDs, which promote the interaction between MDM2 and an E3 ubiquitin ligase, leading to the ubiquitination and subsequent degradation of MDM2. This degradation can then impact the p53 pathway, as MDM2 normally inhibits p53 [66]. Originally derived from the PROTAC MDM2 degrader MD-222, MG-277 does not function as a conventional MDM2-targeting PROTAC. Instead, it exhibits potent, p53-independent anticancer activity by acting as an MGD that induces the degradation of GSPT1, a translation termination factor [67]. This study [67] highlights the unexpected functional switch from targeted MDM2 degradation to GSPT1-mediated protein degradation, demonstrating the potential of structural tuning to create new therapeutic modalities.

X-linked inhibitor of apoptosis (XIAP) and cellular inhibitor of apoptosis protein 1/2 (cIAP1/2) are key members of the inhibitor of apoptosis protein family, which can be effectively degraded using a PROTAC strategy that recruits CRBN. The PROTAC-induced degradation of these XIAPs suppresses TNF α-induced innate immune signaling and inhibits cancer cell migration, invasion, and survival, ultimately leading to apoptotic cell death [68]. Notably, these findings highlight that XIAP and cIAP1/2 are susceptible to ligand-induced degradation mechanisms, suggesting that they may also be degradable by MGDs. Since MGDs also function by promoting neomorphic interactions between an E3 ligase (e.g., CRBN) and target proteins, the degradability of IAPs by PROTACs opens up the possibility of developing MGDs that could selectively degrade XIAP or cIAP1/2 for therapeutic applications in cancer and inflammatory diseases.

Ring Finger Protein 146 (RNF146) is a RING-type E3 ubiquitin ligase that contains a WWE domain, enabling it to bind polyADP-ribose (PAR) and recognize PARylated substrates in a PAR-dependent manner [69]. Identified substrates include Axin1, 3BP2, and PARP1. RNF146 plays key roles in Wnt/β-catenin signaling and the DNA damage response. Notably, RNF146 positively regulates Wnt signaling by promoting the tankyrase-dependent degradation of Axin, a negative regulator of β-catenin. By linking tankyrase-mediated PARsylation to ubiquitin-dependent degradation, RNF146 functions as a PARsylation-directed E3 ligase, establishing a mechanistic bridge between PAR signaling and proteasomal degradation [70].

Deltex1 (DTX1) is a RING-type E3 ubiquitin ligase that plays a central role in the Notch signaling pathway, where it mediates the ubiquitination and degradation of Notch intracellular domains. Functionally, it exhibits characteristic RING E3 ligase features, enabling it to interact with E2 enzymes and target proteins to catalyze polyubiquitination, including K48- and K63-linked ubiquitin chains [71]. This mechanism allows DTX1 to regulate both proteasomal degradation and non-degradative signaling events. Beyond its canonical role in Notch receptor turnover, DTX1 functions independently of classical Notch signaling by interacting with the transcriptional coactivator p300 in the nucleus; this interaction represses MASH1 transcription and subsequently blocks neural progenitor cell differentiation [72,73]. Despite its diverse biological functions, DTX1 has not yet been utilized as an E3 ligase in MGDs strategies, and no small molecules are currently known to induce neomorphic interactions between DTX1 and non-native substrates. However, given its established E3 ligase activity, substrate flexibility, and involvement in critical signaling pathways, DTX1 represents a compelling theoretical candidate for future MGD development. Further structural or chemical biology studies may reveal ligand-binding sites or conformational surfaces that could be leveraged to engineer small molecules capable of redirecting DTX1’s ubiquitin ligase activity toward disease-relevant targets. Thus, while DTX1 is not yet an established MGD platform, it remains a promising and underexplored target within the TPD landscape.

Ubiquitin protein ligase E3 component n-recognin 7 (UBR7) is a putative E3 ligase that covalently binds to manumycin compounds through their C374 in breast cancer cells [74]. This binding represents a specific interaction between the natural product and the ligase. It leads to an MGD interaction with the tumor suppressor TP53, acting as a neosubstrate. The resulting interaction triggers transcriptional activation of p53, which subsequently leads to cell death in breast cancer cells. In essence, the study reveals a new anticancer mechanism for the manumycin natural product family [74]. It also emphasizes the significant potential of combining chemoproteomics with multicovalent natural products as a powerful strategy for identifying and developing new MGD therapeutics.

Ongoing research continues to uncover additional E3 ligases with unique substrate specificities and therapeutic implications, further broadening the scope of MGD development [54] (Table 1).

**Table 1:** Summary for E3 ligases beyond the DCAF family.

E3 Ligase	Function/Role & Key Mechanism	Status/Therapeutic Potential	Citing Publications
**VHL**	A component of the VHL-Cullin-RING E3 ligase complex. Small molecules bind to a specific pocket on VHL and induce a new, “neomorphic” interaction with proteins like **CDO1**, leading to their degradation.	**Established MGD Platform:** Its mechanism has been validated at the atomic level through X-ray crystallography.	[65]
**MDM2**	An E3 ligase that normally targets p53 for degradation. A specific MGD (**MG-277**) was found to have a functional switch, instead targeting the translation factor **GSPT1** for degradation in a p53-independent manner.	**Established MGD Platform:** Demonstrates how structural modifications can create new therapeutic modalities with unexpected substrate specificity.	[67]
**XIAP & cIAP1/2**	Key members of the inhibitor of apoptosis protein (IAP) family. While known to be degraded by PROTACs that recruit CRBN, this susceptibility to ligand-induced degradation suggests they could also be targeted by MGDs.	**Promising Candidates:** Susceptible to ligand-induced degradation, making them strong candidates for future MGD development.	[68]
**RNF146**	A RING-type E3 ligase containing a **WWE domain** that recognizes PARylated substrates. It links tankyrase-mediated PARsylation to ubiquitin-dependent degradation of proteins like **Axin**.	**Research Candidate:** A unique, PAR-dependent mechanism that could be leveraged for future MGDs.	[69,70]
**DTX1**	A RING-type E3 ligase that regulates the Notch signaling pathway. It can target Notch intracellular domains for degradation, but no MGDs are currently known to engage it.	**Underexplored Candidate:** A promising theoretical target for MGD development due to its established E3 ligase activity and involvement in key signaling pathways.	[71,72,73]

Note: VHL, Von Hippel-Lindau; CDO1, cysteine dioxygenase 1; MGD, Molecular glue degrader; MDM2, Mouse double minute 2; GSPT1, G1 to S phase transition 1; XIAP, X-linked inhibitor of apoptosis protein; cIAP1/2, cellular inhibitor of apoptosis protein 1/2; PROTACs, proteolysis-targeting chimeras; CRBN, cereblon; RNF146, Ring Finger Protein 146; WWE, Tryptophan-Tryptophan-Glutamate resi-dues; PARylated, poly-ADP-ribosylated; DTX1, Deltex1.

### 2.5 Expanding Beyond CRBN: Broadening the Horizon of E3 Ubiquitin Ligases

The advent of TPD represents a paradigm shift in pharmacology, offering a potent strategy to eliminate disease-causing proteins previously considered undruggable by conventional small-molecule inhibitors [75]. Among the primary drivers of this approach are MGDs, which, alongside PROTACs, hijack the cell’s natural protein disposal machinery, the ubiquitin-proteasome system. At the core of this process are E3 ubiquitin ligases, acting as crucial mediators that tag a target protein for degradation [23].

A significant challenge in the TPD field particularly regarding MGDs, is the underutilization of the vast E3 ligase family. Nearly all clinical and preclinical degraders overwhelmingly rely on a very small, well-characterized subset, most notably CRBN [76]. This heavy reliance on a limited number of E3 ligases imposes several critical constraints on the full potential of MGDs. First, it restricts the range of proteins that can be effectively degraded. Successful degradation requires the formation of a stable and productive ternary complex between the E3 ligase and the POI. However, many disease-relevant proteins are structurally or spatially incompatible with CRBN-mediated proximity, rendering them inaccessible to current MGD strategies [77]. 

Second, the ubiquitous expression of CRBN across diverse human tissues, while beneficial for some applications, significantly hinders the achievement of tissue- or cell-type specificity. For diseases requiring selective action within particular organs or cell populations, this broad expression can lead to on target but off-tissue toxicity, limiting the development of safer, more precise MGD therapeutics [78,79]. In contrast, exploiting E3 ligases with restricted or disease-specific expression patterns holds promise for enhancing selectivity and therapeutic index [79].

Third, as MGD-based therapies advance into clinical use, the emergence of acquired resistance presents a growing challenge. Cancer cells, for example, can escape degradation pressure by downregulating or mutating the recruited E3 ligase, such as CRBN, ultimately leading to therapeutic failure [77]. Expanding the repertoire of druggable E3 ligases would provide crucial alternative options to counteract these resistance mechanisms and sustain clinical efficacy. Studies using clustered regularly interspaced short palindromic repeats (CRISPR)-suppressor scanning and haploid genetics have revealed that resistance to MGDs targeting neosubstrates, such as G1 to S phase transition 1 (GSPT1) and RBM39, which interact with CRBN and DCAF15, respectively, is directly linked to mutations altering the ternary complex heterodimerization surface [77]. These hotspot mutations, validated in patients who relapse from degrader treatment, disrupt the intended degradation process [80]. Several studies have reported loss-of-function mutations or reduced expression of E3 ligase substrate receptors as a major pathway of resistance to degraders. This mechanism is exemplified by the connection between IKZF1/3 and CRL4^CRBN^ E3 ubiquitin ligase mutations and resistance to immunomodulatory drugs in multiple myeloma, which is consistent with the positive correlation of CRBN expression levels with response to lenalidomide and pomalidomide in multiple myeloma patients [81,82].

Research in the field is currently focused on multiple strategies to explore and recruit novel E3 ligases. These efforts include the systematic, multiomics characterization of the entire E3 ligase family to rank individual ligases based on their chemical ligandability, expression patterns, and known PPIs [16]. Advanced discovery platforms are guiding this rational discovery process, ranging from chemoproteomic covalent screening techniques that identify new ligandable “hotspots” on E3 surfaces [83] to computational deep-learning (DL) models capable of predicting degrons and E3-substrate binding interfaces [84]. This approach necessitates a deeper understanding of E3 ligase biology, encompassing factors such as substrate selection modes, catalytic specificity, regulatory mechanisms, expression dynamics, and physiological functions [85].

Despite being smaller and simpler molecules (typically <500 Da) than PROTACs-thereby offering superior cellular permeability and the potential for oral administration-designing effective MGDs remains highly complex. This complexity arises from the unpredictable nature of the PPIs they must modulate [10]. Consequently, many MGDs have been discovered serendipitously, and their development continues to rely on innovative screening methods to identify compounds that can effectively stabilize or induce these interactions [10]. While the current MGD landscape is constrained by its heavy dependence on a narrow set of E3 ligases, this limitation simultaneously represents the field’s greatest opportunity. By developing innovative strategies to engage the vast, untapped majority of the E3 ligase family, researchers can significantly expand the degradable proteome, overcome therapeutic resistance, and design the next generation of highly specific and potent MGDs [10,86].

### 2.6 Characteristics of an Ideal E3 Ligase for MGD Development

An ideal E3 ligase for MGD development would possess characteristics that enable efficient and selective protein degradation. Key features include strong and specific binding to the target protein, the ability to induce ubiquitination and subsequent proteasomal degradation, and a favorable pharmacological profile for drug development [86,87,88].

**Figure 5:**
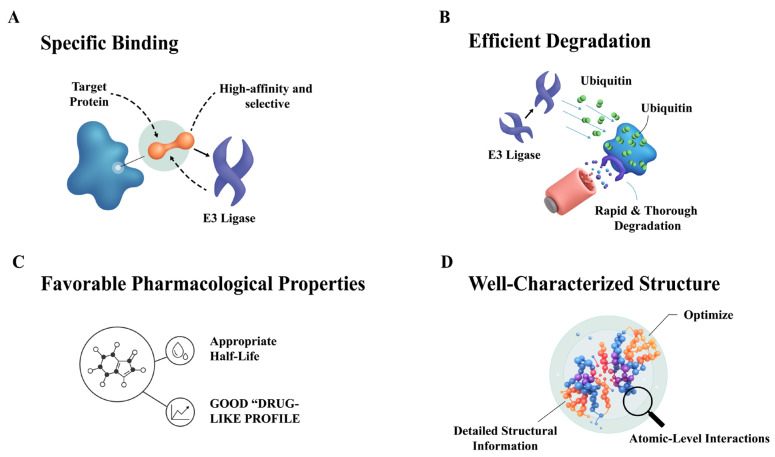
Characteristics of an Ideal E3 Ligase for MGD Development. (**A**) Specific Binding. The degrader must exhibit high affinity and selectivity, enabling it to specifically bind to both the target protein and the E3 ligase. (**B**) Efficient Degradation. The strategy must ensure the effective and efficient transfer of ubiquitin to the target protein, leading to its rapid and thorough degradation by the proteasome. (**C**) Favorable Pharmacological Properties. The molecule should possess a good “drug-like profile”, including properties that are beneficial for clinical development, such as bioavailability and appropriate half-life. (**D**) Well-Characterized Structure. Detailed structural information of the degrader, its target, and the E3 ligase is essential to understand and optimize the atomic-level interactions that drive the degradation process. The pictures were drawn using Illustrator. Note: MGD, Molecular glue degrader.

When selecting an ideal E3 ligase for MGD development, several key criteria must be considered:

First, specificity is essential. The E3 ligase should exhibit high affinity and selectivity for the target protein, crucial for minimizing off-target effects and maximizing the desired degradation. It must also be able to recognize and bind to specific degradation signals (degrons) on the target protein [87,89].
Second, the E3 ligase must demonstrate efficient ubiquitination and degradation. It should effectively transfer ubiquitin from an E2 ubiquitin-conjugating enzyme to the target protein, initiating the degradation pathway [88,90]. Crucially, the ubiquitinated target protein must then be recognized and degraded by the proteasome, the cellular machinery responsible for protein breakdown.
Third, favorable pharmacological properties are vital. The E3 ligase should possess a good drug-like profile, including appropriate molecular weight, lipophilicity, solubility, and a lack of reactive groups or pan-assay interference compounds [87,91]. Furthermore, well-characterized structural information regarding how the E3 ligase interacts with its binding partners can significantly facilitate the rational design of MGDs with improved potency and selectivity [91].
Finally, additional considerations include the E3 ligase class and its regulatory mechanisms. Different E3 ligase families (e.g., RING, HECT, or RBR) operate through distinct mechanisms, and the choice of E3 ligase can significantly influence the design and efficacy of MGDs [37]. Understanding how E3 ligases are regulated (e.g., through post-translational modifications or interactions with other proteins) can also provide invaluable insights into optimizing their activity for successful MGD development [90,92] (Fig. 5).

RING, HECT, and RBR constitute mechanistically and structurally diverse enzyme families that orchestrate the selective ubiquitination and degradation of proteins. Understanding their catalytic modes is fundamental for therapeutic exploitation: RING ligases (e.g., CRLs, UBR family) act as scaffolds that facilitate direct ubiquitin transfer from E2 to the substrate; HECT ligases (e.g., NEDD4, UBE3A) form a transient thioester intermediate allowing tighter catalytic control; and RBR ligases (e.g., Parkin, HOIP) integrate features of the two former groups, allowing hybrid regulation. In TPD and MGD development, E3 ligases form molecular hubs that dictate substrate specificity and degradation efficiency. However, current therapeutics rely heavily on a narrow subset, mainly CRBN, which limits tissue selectivity and leads to potential resistance. Expanding the usable E3 repertoire, including DCAF15, DCAF16, or other context-specific ligases (MDM2, RNF146, DTX1, UBR7), is key to diversifying degrader strategies and accessing undruggable targets.

An ideal E3 ligase for MGD applications should combine substrate selectivity, efficient ubiquitin transfer, structural tractability, and favorable pharmacological properties. Integrating structural biology, chemoproteomics, and computational design allows the rational engagement of new E3 ligases, transforming the conceptual understanding of E3 enzymology into practical frameworks for next-generation therapeutic development.

## 3 Representative MGD-E3 Ligase-Substrate Pairs: Bridging Molecular Discovery and Translational Outcomes

This rapidly advancing field of drug discovery encompasses various MGDs and their corresponding protein targets. These examples illustrate how small molecules can induce or enhance the interaction between E3 ubiquitin ligases and specific substrates, triggering ubiquitination and subsequent proteasomal degradation. By categorizing MGDs based on their target proteins and E3 ligases, these cases reveal the mechanistic diversity and therapeutic potential of TPD. Notably, they constitute a bridge between the molecular mechanisms of E3-substrate modulation and their translational impact in disease treatment.

The first mechanistic framework emerged from discovering GSPT1/CRBN MGDs, including CC-885, CC-90009, and SJ6986, which suppress translation termination and induce apoptosis through the selective degradation of GSPT1 [93,94,95,96]. These molecules, some discovered rationally and others serendipitously during PROTAC optimization, exemplify how discoveries at the molecular level can evolve into clinically relevant therapeutics (e.g., CC-90009 has progressed into clinical trials for acute myeloid leukemia). Similarly, IKZF1/3-targeting CRBN MGDs, including thalidomide, lenalidomide, and pomalidomide, represent the most successful translational paradigm, converting an initially teratogenic compound into a cornerstone of multiple myeloma therapy through selective CRBN-dependent degradation of transcription factors [97,98,99,100]. Mechanistically elucidating the degradation of Spalt Like Transcription Factor 4 (SALL4) and p63 further linked the molecular basis of thalidomide-induced teratogenicity to structure-guided analog design, supporting the development of non-teratogenic CRBN modulators [101,102].

In addition to the CRBN system, the utilization of alternative E3 ligases has significantly expanded the translational reach of MGDs. For instance, compounds such as NRX-252114 and NRX-252262 enhance the natural interaction between mutant β-catenin and its endogenous ligase, SCF^βTrCP^. This approach offers a novel means to target transcriptional regulators that were once considered “undruggable” [53,103]. Similarly, MGDs like WBC100 recruit the CHIP ligase to degrade the oncogenic transcription factor c-Myc. Furthermore, aryl sulfonamides, such as E7820 and indisulam, recruit DCAF15 to induce the degradation of RBM39. These examples demonstrate how induced neomorphic interactions can achieve potent antitumor effects through diverse ligase-substrate architectures [104,105,106,107,108]. Moreover, next-generation CRBN modulators, including CC-3060 and CC-647, have been shown to promote the degradation of ZBTB16, suggesting new therapeutic strategies for acute promyelocytic leukemia [109]. Collectively, these advancements illustrate the expanding landscape of MGDs in targeting diverse target proteins through the recruitment of various E3 ubiquitin ligases.

The expanding chemical and mechanistic diversity of MGDs continues to blur the line between traditional ligand-target pharmacology and induced proximity biology. Molecules inspired by natural products, such as asukamycin and related maremycins, covalently modify UBR7, leading to the degradation of p53 [74]. In contrast, CR8 and CT7439 exploit the CDK12-Cyclin K complex as an adaptor for DDB1-dependent degradation and have advanced into clinical evaluation [110]. Covalent MGDs, such as EN450, further diversify this landscape by establishing proximity between the E2 enzyme UBE2D (UbcH5) and NFKB1, driving selective degradation through noncanonical ubiquitination [111]. Natural products, such as bufalin, demonstrate yet another pathway, promoting E2F transcription factor 2 (E2F2) degradation via zinc finger protein 91 (ZFP91), and illustrate how bioactive compounds from traditional medicine can yield mechanistically novel degradation pathways with therapeutic potential [112].

Other distinctive examples underscore the versatility of the MGD concept: WB214 induces the dual degradation of MDM2 and p53 via CRBN [113]; BI-3802 promotes BCL6 polymerization, thereby enabling SIAH1-mediated degradation [114]; and DNMDP stabilizes the PDE3A-SLFN12 complex, enhancing RNase activity and inducing apoptosis independently of the proteasome [115]. Expanding beyond classical ubiquitin-mediated turnover, HB007 recruits CUL1 and F-box protein 42 to CAPRIN1, leading to the degradation of SUMO1. This represents a pioneering example of SUMO-targeting molecular glues that broaden the conceptual boundaries of induced degradation [116].

Overall, these representative pairs exemplify the continuum between molecular discovery and translational medicine. From the structural characterization of ternary complexes (e.g., CRBN-IKZF, DCAF15-RBM39) to the clinical progression of optimized candidates (e.g., CC-90009, CT7439), these studies highlight how mechanistic insights directly inform drug design, selectivity engineering, and safety evaluation (Table 2).

**Table 2:** Representative MGD-E3 ligase-substrate pairs identified to date.

Target/E3 Ligase	MGD(s)	Mechanism of Action	Citing Publications
GSPT1/CRBN	CC-885, ZXH-1-161, CC-90009, SJ6986, MI-389, MG-277, GBD9	Induce CRBN-dependent degradation of GSPT1. GBD9 is a dual-target MGD/PROTAC for GSPT1 and BTK.	[67,93,94,95,96]
IKZF1/3/CRBN	Thalidomide, Lenalidomide, Pomalidomide, CC-220, CC92480	Induce CRBN-dependent degradation of the transcription factors IKZF1 and IKZF3.	[26,32,97,98,100]
SALL4/CRBN	S-thalidomide, Pomalidomide, Lenalidomide	Induce CRBN-dependent degradation of SALL4, a mechanism linked to their teratogenic effects.	[101]
p63/CRBN	Thalidomide	Acts as a CRBN-dependent molecular glue to degrade p63 isoforms, which provides another reason for its teratogenic effect.	[102]
β-catenin/SCF β-TrCP	NRX-252114, NRX-252262	Enhance the interaction betweenβ-catenin and its natural E3 ligase SCF β-TrCP, promoting its degradation.	[53,103]
cMyc/CHIP	WBC100	Induces the degradation of cMyc via the CHIP E3 ligase, leading to cancer cell apoptosis.	[104]
eRF1/Ribosomal Subunit	SRI-41315	Anchors the eRF1 protein to the ribosome, leading to its degradation via ribosome-associated quality control.	[117,118]
RBM39/DCAF15	E7820, Indisulam, CQS (NSC 339004)	Induce CRL4-DCAF15-mediated ubiquitination and degradation of RBM39. Arylsulfonamides bind to a shallow pocket on DCAF15 to enable this interaction.	[105,106,107,108]
ZBTB16/CRBN	CC-3060, CC-647	Promote the degradation of ZBTB16 via distinct CRBN-dependent mechanisms offering a new therapeutic strategy.	[109]
p53/UBR7	Maremycin polyketides (e.g., asukamycin)	Target C374 of the E3 ligase UBR7, inducing it to ubiquitinate and degrade the tumor suppressor TP53.	[74]
Cyclin K (CDK12)/DDB1	HQ461, CR8, CT7439	Induce the formation of a complex between the CDK12-Cyclin K complex and the CUL4-DDB1 ligase, leading to Cyclin K degradation.	[110]
NFKB1/UBE2D (E2)	EN450	Covalently interacts with the E2 enzyme UbcH5, uniquely inducing proximity to the oncogenic transcription factor NFKB1 for its degradation.	[111]
E2F2/ZFP91	Bufalin	Acts as an MGD to facilitate the formation of a ternary complex, promoting E2F2 ubiquitination and degradation.	[112]
MDM2/CRBN	WB214	Induces the degradation of both MDM2 and p53, with MDM2 acting as a novel substrate for CRBN.	[113]
BCL6/SIAH1	BI-3802	Binds to the BTB domain of BCL6, inducing its aggregation and subsequent ubiquitination and degradation by the SIAH1 E3 ligase.	[114]
PDE3A/SLFN12	DNMDP	Strengthens the interaction between PDE3A and SLFN12, enhancing SLFN12’s RNase activity and inducing apoptosis.	[115]
CAPRIN1/CUL1	HB007	Selectively binds to CAPRIN1, recruiting the F-box protein 42/CUL1 E3 ligase, leading to the ubiquitination and degradation of SUMO1.	[116]

Note: GSPT1, G1 to S phase transition 1; CRBN, cereblon; GBD9, GTP-binding domain-containing protein 9; BTK, Bruton’s tyrosine kinase, IKZF, IKAROS family zinc finger; SALL4, Spalt Like Transcription Factor 4; SCF β-TrCP, Skp1-Cullin-F-box β-transducin repeat containing E3 ubiquitin protein ligase; CHIP; Carboxy-terminus of Hsc70-interacting protein; eRF1, Eukaryotic translation termination factor 1; RBM39, RNA-binding protein 39; DCAF15, DDB1 And CUL4 Associated Factor 15; ZBTB16, Zinc finger and BTB domain-containing protein 16; UBR7, Ubiquitin Protein Ligase E3 Component N-Recognin 7; CDK12, Cyclin-Dependent Kinase 12; DBB1, Damage Specific DNA Binding Protein 1; NFKB1, Nuclear Factor Kappa B Subunit 1; UBE2D, Ubiquitin-conjugating enzyme E2 D; E2F2, E2F transcription factor 2; ZFP91, zinc finger protein 91; MDM2, Mouse Double Minute 2 homolog, BCL6, B-cell lymphoma 6 protein; SIAH1, Siah E3 ubiquitin protein ligase 1; PDE3A, Phosphodiesterase 3A; SLFN12, Schlafen Family Member 12; DNMDP, 6-(4-(diethylamino)-3-nitrophenyl)-5-methyl-4,5-dihydropyridazin-3(2H)-one; CAPRIN1, Cell cycle associated protein 1; CUL1, Cullin 1 SUMO1, Small Ubiquitin-like Modifier 1.

Next, we will bridge the molecular and translational aspects of MGD research, exploring how a deeper understanding of structure and mechanism can directly impact therapeutic innovation.

High-resolution structural and biochemical analyses, such as those of CRBN-IKZF and DCAF15-RBM39 complexes, have accelerated translation by enabling immediate structure-activity relationship modeling, providing crucial information for safety profiling (e.g., avoidance of unintended SALL4 or p63 recruitment) and facilitating streamlined progression from molecular glue identification to optimized clinical candidates [32,101,102,103,104,105,106,107,108,117,118]. 

Mechanistically, MGDs can operate through multiple routes: they may create entirely new E3-substrate interfaces, enhance native degron recognition, induce target aggregation that exposes hidden degrons, covalently reprogram ubiquitination enzymes like E2 or E3, or stabilize enzymatic complexes that elicit nondegradative but cytotoxic effects. Each of these mechanistic pathways carries unique translational implications for optimizing selectivity, identifying predictive biomarkers, and managing potential toxicities [53,74,111,114,115]. The discovery process itself often depends on serendipity and cross-platform exploration; many MGDs have been identified unexpectedly during PROTAC optimization campaigns or broad phenotypic screens. This highlighting the value of maintaining diverse and interdisciplinary discovery pipelines to yield therapeutically meaningful glues [67,119]. Equally crucial is the thorough assessment of substrate specificity and safety. For instance, the unintended degradation of SALL4 and p63 underscores the critical importance of early neosubstrate profiling to mitigate developmental toxicity and inform the rational design of safer, more selective analogs [101,102]. 

Collectively, these representative MGD-E3-substrate triplets provide concrete mechanistic connections between discovery-level biology-including structural assembly, ternary complex formation, and ubiquitin linkage specificity-and translational outcomes, such as the advancement of clinical candidates, biomarker-guided development, and rational safety engineering. Together, they exemplify a rapidly maturing paradigm in which small molecules reprogram the ubiquitin-proteasome system to achieve precise and disease-relevant control of target proteins.

## 4 Significant Clinical Trials and Promising Therapeutic Outcomes Associated with MGD as an Anticancer Agent

Building upon the mechanistic diversity of E3 ligases described in Section 2, which allows selective and context-specific protein ubiquitination, recent advances have translated these molecular insights into clinically meaningful applications through MGD-based anticancer therapies. By targeting and degrading proteins that are essential for cancer cell survival or proliferation, MGDs can disrupt oncogenic pathways and potentially induce tumor regression. MGDs are generally smaller than other targeted protein degraders, such as PROTACs, often offering better pharmacokinetic properties, including improved absorption, distribution, metabolism, and excretion. Several companies and academic groups are actively advancing MGDs as anticancer therapeutics, with ongoing efforts to optimize their use across various cancer types and clarify their mechanisms of action [17,120,121]. Notably, a fumarate-based MGD handle was developed to target specific kinases associated with cancer [119], while another study highlighted the therapeutic potential of MGDs in multiple myeloma and other hematologic malignancies [122]. Currently, many clinical trials are evaluating the safety, tolerability, pharmacokinetics, and therapeutic potential of these agents in patients with solid tumors, hematologic cancers, and immune disorders.

CRBN E3 ligase modulators (CELMoDs) represent one of the most clinically advanced classes of MGDs. Unlike early IMiDs, CELMoDs are rationally designed to enhance the recruitment and subsequent degradation of specific target proteins, such as AIKZF1 and IKZF3, with greater potency and selectivity. CC-220 (iberdomide) induces IKZF1 and IKZF3 degradation and has exhibited strong therapeutic activity in relapsed/refractory multiple myeloma (RRMM). In the CC-220-MM-001 trial (NCT02773030), combination therapy with bortezomib and dexamethasone achieved an overall response rate (ORR) of 100% in patients newly diagnosed with multiple myeloma, with 87.5% and 56.25% achieving very good partial response or complete response, respectively, including a high rate of minimal residual disease negativity [123]. In patients with refractory RRMM, iberdomide plus dexamethasone demonstrated a 26.2% ORR, with manageable adverse events and preserved life quality, supporting its evaluation in Phase III trials, such as EXCALIBER-RRMM (NCT04975997) [124].

CC-122 (avadomide), another CELMoD, similarly promotes IKZF1/3 degradation and is efficient in non-Hodgkin’s lymphoma (NHL) and multiple myeloma. In a Phase I trial (NCT01421524), avadomide achieved an ORR of 60% in patients with NHLs, and subsequent studies combining it with obinutuzumab demonstrated an ORR of 71%, with 40% complete responses, establishing its recommended Phase II dose and confirming durable responses with manageable safety [125,126,127,128,129,130]. More recently, CC-99282 (golcadomide) has advanced into trials for relapsed/refractory NHL. In the CC-99282-NHL-001 study (NCT03930953), individual therapy achieved an ORR of 43% in heavily treated patients, while follicular lymphoma patients achieved 75% ORR, including 38% complete responses, with a reassuring safety profile [131,132,133].

Beyond CRBN modulators, arylsulfonamide-based degraders, such as indisulam (E7070) and E7820, target the splicing factor RBM39. Indisulam has exhibited therapeutic potential in AML and MDS when combined with chemotherapy, achieving a complete remission rate of 35% in a Phase II trial (NCT01692197); notably, it maintained an acceptable safety profile despite its limited efficacy as a monotherapy [134,135,136]. Similarly, E7820 promotes the degradation of RBM39. Phase II clinical testing of E7820 in myeloid malignancies (NCT05024994) has reported modest ORRs alongside good tolerability [137,138,139]. These findings underscore RBM39 as a promising target protein for splicing factor therapy, although further optimization of these MGDs remains necessary for broader therapeutic applications.

Another highly promising MGD candidate is CC-90009, which binds CRL4^CRBN^ to induce GSPT1 degradation, triggering apoptosis in AML cells. In early clinical testing (NCT02848001), CC-90009 monotherapy rapidly reduced peripheral and bone marrow progenitors in patients with relapsed/refractory AML. Its combination with venetoclax/azacitidine is currently being evaluated in Phase I/II studies (NCT04336982), with completion expected by 2025 [140]. Similarly, MRT-2359, another selective GSPT1 degrader, has entered Phase I/II trials (NCT05546268) for MYC-driven tumors, including lung cancer and diffuse large B-cell lymphoma, highlighting the expanding applications of MGDs beyond hematologic malignancies.

The clinical progress of MGDs, including iberdomide, avadomide, golcadomide, indisulam, E7820, CC-90009, and MRT-2359-demonstrates their capacity to selectively degrade oncogenic target proteins, achieve high response rates in refractory patient populations, and maintain manageable safety profiles. Collectively, these findings underscore the robust therapeutic potential of MGDs, which are steadily advancing from hematologic cancers toward broader applications in solid tumors and other disease indications. This expansion reflects a maturing field where induced protein degradation is increasingly being established as a precise and effective therapeutic modality (Table 3).

**Table 3:** Clinical progress of representative MGDs.

Drug (Code Name)	E3 Ligase/Target Protein	Cancer Type(s)	Trial Phase/Status	Key Outcomes	Citing Publications
Iberdomide (CC-220)	CRL4CRBN/IKZF1, IKZF3	Multiple Myeloma (RRMM, NDMM)	Phase 1/2 (NCT02773030); Phase 3 ongoing (EXCALIBER-RRMM, NCT04975997)	NDMM: ORR 100%, ≥VGPR 87.5%, CR 56.3%, high MRD negativity; RRMM: ORR 26.2%, manageable safety.	[123,124]
Avadomide (CC-122)	CRL4CRBN/IKZF1, IKZF3	Non-Hodgkin’s Lymphoma, Multiple Myeloma	Phase 1/2 (NCT01421524, others)	NHL: ORR 60% (monotherapy); Avadomide + Obinutuzumab: ORR 71%, CR 40%, durable responses, established Phase II dose.	[125,126,127,128,129]
Golcadomide (CC-99282)	CRL4CRBN/IKZF1, IKZF3	Relapsed/Refractory NHL (incl. FL, DLBCL)	Phase 1 (NCT03930953)	Overall ORR 43% (heavily treated); in FL: ORR 75%, CR 38%; favorable safety profile.	[131,132,133]
Indisulam (E7070)	CRL4DCAF15/RBM39	AML, MDS, solid tumors	Phase 2 (NCT01692197)	In AML/MDS with chemo: CR 35%; acceptable safety; limited monotherapy efficacy.	[134,135,136]
E7820	CRL4DCAF15/RBM39	Myeloid Malignancies	Phase 2 (NCT05024994)	Modest ORR, good tolerability; confirms RBM39 as a druggable splicing factor.	[137,138,139]
CC-90009	CRL4CRBN/GSPT1	AML, other hematologic malignancies	Phase 1 (NCT02848001); Phase 1/2 (NCT04336982, ongoing)	Monotherapy: rapid reduction of AML progenitors; combination with Venetoclax/Azacitidine under evaluation.	[140]
MRT-2359	CRL4CRBN/GSPT1	MYC-driven solid tumors (lung cancer, DLBCL)	Phase 1/2 (NCT05546268, ongoing)	Early evidence of clinical activity in MYC-dependent tumors; safety and efficacy evaluation in progress.	NCT05546268

Note: CRL, Cullin-RING Ligase; CRBN, cereblon; IKZF, IKAROS family zinc finger; RRMM, Re-lapsed/Refractory Multiple Myeloma; NDMM, Newly Diagnosed Multiple Myeloma; CR, complete response; ORR, objective response rate; NHL, Non-Hodgkin’s Lymphoma; RBM39, RNA-binding protein 39; AML, Acute Myeloid Leukemia; MDS, Myelodysplastic Syndromes; GSPT1, G1 to S phase transition 1.

## 5 Strategies for Discovering Novel E3 Ligases in MGD Development

Identifying novel E3 ligases is central to expanding the therapeutic scope of MGDs. Multiple complementary strategies-encompassing phenotypic screening, multiomics technologies, biophysical tools, and computational modeling-are currently being employed to uncover previously uncharacterized E3 ligases and their interactions with neosubstrates. Strategies aimed at discovering new E3 ligases for TPD involve expanding the available toolbox beyond the currently exploited ligases, enhancing target selectivity, and overcoming potential resistance mechanisms. These objectives can be achieved through various approaches, including the identification of new E3 ligase ligands, a deeper understanding of their underlying mechanisms, and the strategic integration of these insights into the design of MGDs.

### 5.1 Phenotypic Screening Approaches

Cell-based phenotypic screens are highly effective in identifying molecules that induce protein degradation, even without prior knowledge of the specific E3 ubiquitin ligase involved. These screens use functional readouts or reporter systems to detect changes in cellular behavior or protein levels resulting from protein degradation [141,142].

One well-defined process for identifying potential degraders involves cell line preparation followed by high-throughput degradation assays [141]. For instance, researchers engineered HeLa cell lines to stably express HaloTag-GFP, alongside a control line harboring a HaloTag D106A mutation to counter-screen for false positives. Test com-pounds were dispensed into 384-well plates, and after two days of incubation, GFP fluorescence was measured to quantify protein degradation. Compounds that induced over 50% degradation of HaloTag-GFP, while causing less than 50% degradation of the D106A mutant, were identified as hits [141]. Alternatively, these assays can utilize cellular responses or reporter systems affected by protein degradation rather than direct protein level measurements. This approach may involve monitoring a decrease in the activity of a specific protein or employing a fluorescent protein fused to the target protein to track its degradation in real-time.

Another multistep approach for phenotypic screening incorporates the production of lentiviral particles, the establishment of stable cell lines, and flow cytometry-based screening [143]. Lentiviral virions encoding a GFP-tagged POI are produced and transduced into HeLa S3 cells. Cells exhibiting stable expression are selected and transfected with plasmids encoding various biodegraders-consisting of E3 ligases linked to a specific protein binder. The degradation of the POI is assessed by measuring GFP fluorescence intensity via flow cytometry. Hits are subsequently confirmed through retransfection and validated for proteasome-dependent degradation using specific inhibitors [143].

While phenotypic screens can identify molecules responsible for protein degradation, they do not initially identify the specific E3 ligase involved. Further experiments, often using target deconvolution strategies, become necessary [64]. For example, CR8, initially identified as a CDK inhibitor, was later found to be an MGD that degrades cyclin K. Researchers used drug resistance and reporter-based CRISPR screens to identify DDB1, an E3 ligase adaptor protein, as the key player in the degradation of CCNK [64]. Similarly, HQ461 was identified as an antitumor agent through a genome-wide CRISPR drug resistance screen, revealing the power of phenotypic screens in uncovering the mechanism of action of MGDs [64]. Site-specific ligand-incorporation-induced proximity technology enables the incorporation of unnatural amino acids into proteins, allowing the identification of PROTACable sites and facilitating the discovery of new TPD effector ligands [144].

CRISPR-based E3 ligase knockout/knockdown screens are systematically used to identify the E3 ligases required for MGD-induced degradation of specific substrates [38,145]. This method involves creating a comprehensive small guide RNA (sgRNA) library targeting over 700 human E3 ligases and DUBs, including newly designed sgRNAs targeting specific functional domains to enhance gene loss-of-function efficiency [38,145]. HAP1 cell lines stably expressing doxycycline-inducible Cas9 are infected with the sgRNA library, and gene knockout/knockdown is initiated [145]. Cells are cultured with or without selected MGD compounds, and each sgRNA’s relative abundance is measured by next-generation sequencing. Statistical analysis identifies E3 ligases whose ablation impacts MGD-induced degradation. CRISPR-based E3 ligase knockout/knockdown screens are validated with complementary experiments, including competitive growth assays, flow cytometry, cell cycle analysis, immunofluorescence, and western blot [146]. This robust approach is further validated through *in vitro* and *in vivo* experiments, which explore the functional significance of E3 ligases in complex biological contexts, such as tumor growth and immune modulation. Further insights into cellular and immunological roles are gained through additional assays, including western blot, flow cytometry, RNA-seq, ATAC-seq, ChIP-seq, and single-cell RNA-seq, all supported by advanced bioinformatics tools.

### 5.2 Proteomics-Driven Approaches

Quantitative proteomics is a powerful tool for understanding how MGDs influence protein degradation and the role of E3 ligases in this process. Methods such as tandem mass tag (TMT) mass spectrometry (MS), stable isotope labeling by amino acids in cell culture (SILAC), and label-free quantification (LFQ) with data-dependent and data-independent acquisition are employed to globally monitor protein degradation patterns [147]. These approaches accurately quantify protein expression across various samples, allowing researchers to measure protein degradation rates and observe how MGDs impact protein turnover. For example, the isotope-coded affinity tags method involves extracting proteome samples separately before stable isotope labeling, whereas the SILAC method labels proteomes within cells before extraction. Both methods rely on enzymatic digestion and MS for quantification and identification [147].

Subcellular proteomics facilitate the visualization of the spatial expression of E3 ligases and POIs within organelles, further pinpointing the involvement of specific E3 ligases. Additionally, ubiquitinome analysis is essential for identifying specific ubiquitination sites modified by degraders, thereby providing a direct link between MGDs and E3 ligase activity. Proximity labeling-based MS methods can also elucidate degrader-induced PPIs, offering critical insights into E3 ligase recruitment. While isobaric labeling techniques, such as tandem mass tags (TMT), offer significant advantages for multiplexing, achieving reliable quantification requires meticulous attention to every experimental step [148].

By combining quantitative proteomics with methods that enrich for ubiquitinated proteins, researchers can identify proteins that undergo ubiquitination and subsequent degradation in response to MGDs. This approach potentially implicates specific E3 ligases in the degradation process [149]. If a specific E3 ligase is suspected to be involved, its activity can be suppressed through siRNA-mediated knockdown or entirely eliminated via CRISPR-Cas9 knockout. The resulting alterations in protein degradation patterns are then monitored through quantitative proteomics [149,150]. Ultimately, specific E3 ligases are often associated with distinct protein degradation profiles; thus, observing changes in these patterns following the manipulation of E3 ligase activity provides critical insights into the ligase’s physiological function and its target proteins [4,149].

Ubiquitin-remnant profiling, thermal proteome profiling (TPP), and chemical proteomics are complementary techniques used to study protein ubiquitination [151,152,153]. Ubiquitin-remnant profiling (ubiquitome analysis) identifies ubiquitination sites on proteins [154,155]. It involves analyzing peptides generated after trypsin digestion, specifically looking for ubiquitin remnants, providing information on which proteins are ubiquitinated and at which specific sites. TPP identifies protein interactions by detecting changes in their thermal stability [156,157]. When proteins interact with MGDs, their thermal stability can be altered, providing insights into direct or indirect interactions between MGDs, ligases, and their substrates by observing shifts in protein melting temperatures. Chemical proteomics identifies the protein targets of small molecules, including ligases and their substrates [153,154]. This technique uses specialized small-molecule probes (covalent or affinity-based) to capture and identify specific proteins that have interacted with the probe, offering crucial insights into the direct interactors of E3 ligases and their potential substrates.

### 5.3 Genomic and Transcriptomic Approaches

Genome-wide CRISPR-Cas9 screens serve as powerful tools for identifying genes that modulate cellular sensitivity or resistance to MGDs [145,158,159]. These screens employ the CRISPR-Cas9 system to knock out genes on a large scale, allowing researchers to observe subsequent changes in cellular behavior. This systematic approach can identify critical genes, such as E3 ligases and their essential cofactors, that influence therapeutic sensitivity to MGDs.

For example, in a study of glioblastoma (GBM), an E3 ligase sgRNA library was constructed to screen for genes affecting glioma cell growth. RNF185 was identified as a significantly depleted gene, correlating with decreased expression and favorable prognostic significance in patients. *In vitro* overexpression experiments further revealed that RNF185 functions as a tumor suppressor [159]. This observation highlights how genome-wide CRISPR-Cas9 screens can pinpoint specific E3 ubiquitin ligases whose inactivation alters cellular responses to various MGDs [145]. The experimental process involves constructing an extensive sgRNA library, packaging it into lenti-viral vectors, and transducing it into HAP1-Cas9 cells. Pooled CRISPR-Cas9 screens then track cell fitness in the presence or absence of specific small-molecule compounds. High-throughput sequencing of genomic DNA enables the precise quantification of changes in sgRNA abundance. These data undergo rigorous statistical analysis to identify genes whose loss significantly modifies the cellular response to the target protein degradation process [159,160,161].

RNA-seq serves as a powerful tool for uncovering transcriptional alterations associated with MGD treatment, potentially highlighting novel E3 ligases or affected signaling pathways. For instance, in Huntington’s disease, a proteogenomics approach coupled with alternative splicing analysis revealed widespread neuronal differentiation stage- and CAG length-dependent splicing changes. These findings improved the understanding of genes related to RNA processing, neuronal function, and epigenetic modifications associated with mutant HTT splicing [162]. Furthermore, RNA-seq analysis directly facilitates the development and mechanistic understanding of MGDs and other targeted protein de-graders. A systematic characterization of underexplored E3 ligases across seven dimensions-including chemical ligandability, expression patterns, and protein-protein interactions (PPIs)-through the analysis of 30 large-scale datasets led to the identification of 76 E3 ligases as promising candidates for degrader development [16]. In cancer research, developing new degraders like MDEG-541, a MYC PROTAC, has led to CRBN-dependent degradation of relevant cancer targets, implicitly relying on the understanding of target expression and E3 ligase activity often gleaned from RNA-seq data [163].

**Figure 6:**
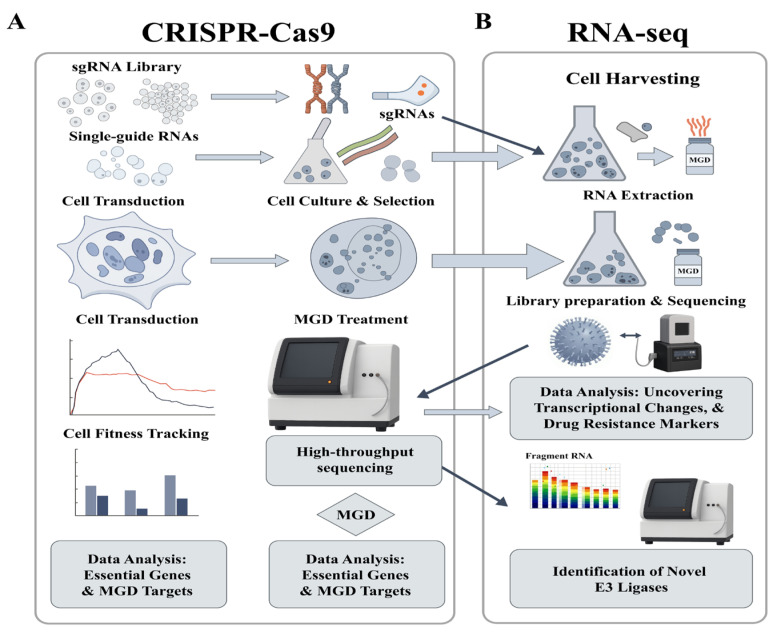
Genome-wide CRISPR-Cas9 screening and RNA-seq analysis for identifying MGD targets and E3 ligases. (**A**) CRISPR-Cas9-based genome-wide screening workflow. A pooled single-guide RNA (sgRNA) library is transduced into cells, followed by culture, selection, and MGD treatment. Cell proliferation and fitness are tracked under MGD exposure, and high-throughput sequencing is performed to analyze essential genes and candidate MGD targets. (**B**) Transcriptomic profiling by RNA-seq. Cells treated with MGDs are harvested for RNA extraction, library preparation, and sequencing. Transcriptome-wide data analysis reveals differential gene expression, transcriptional changes, and drug resistance markers, facilitating the identification of novel E3 ligases involved in MGD-mediated degradation. The pictures were drawn using Illustrator. Note: CRISPR, clustered regularly interspaced short palindromic repeats; MGD, Molecular glue degrader.

RNA-seq is also central to elucidating drug resistance mechanisms related to E3 ligases. In AML, transcriptome sequencing of wild-type and adriamycin-resistant HL60 cells identified differentially expressed genes associated with E3 ubiquitin ligases. This led to the development of a prognostic model comprising five genes, where high expression of UBE2L3 was identified as a reliable biomarker for drug resistance and poor prognosis in AML [164]. Similarly, in cervical cancer (CC), public databases providing mRNA expression and clinical patient data were utilized to develop a robust risk prediction model involving E3 ligase-associated genes, suggesting that targeting specific E3 ligases could be an efficient therapeutic strategy for CC [165] (Fig. 6).

In the context of GBM, analyzing RNA sequencing databases identified specific transcriptomic signatures. For instance, elevated expression of *RNF7*, *TCEB1*, *SOCS1*, and *SOCS3*, which encode components of the Cullin5-RING E3 ligase, was found to predict unfavorable GBM prognoses [166]. Recent advancements in MGDs, which leverage the CRBN E3 ubiquitin ligase to degrade GSPT1, emphasize the importance of understanding the cellular context for selective targeting, including the correlation between therapeutic response and cellular characteristics, such as CRBN expression, often assessed through transcriptomic analyses [167]. Beyond human diseases, the RNA-seq principles for E3 ligase characterization are observed in studies conducted in the halophyte *Sesuvium verrucosum* in response to salinity stress, providing a methodological blueprint for analyzing E3 ligase expression profiles under various conditions, including drug treatments [168].

### 5.4 Biophysical and Biochemical Approaches

High-throughput binding assays, including microscale thermophoresis (MST), surface plasmon resonance (SPR), and amplified luminescent proximity assay (AlphaLISA), are used to screen for direct interactions between MGDs, ligases, and substrates [169,170,171]. These methods rapidly assess binding affinities and kinetics, essential for understanding biological pathways and drug discovery.

MST measures the movement of molecules along a microscopic temperature gradient. Changes in thermophoretic movement upon binding allow for quantification of binding affinity [172,173]. MST is an immobilization-free technique that requires small sample volumes, making it suitable for precious or limited materials and versatile for various biological interactions [171,172,174,175].

SPR is a label-free technique that measures binding events by detecting changes in refractive index near a gold surface as molecules bind [176,177]. It provides real-time information on binding kinetics, including association and dissociation rates, and can be used in a high-throughput array format [176,178]. SPR is widely used in drug discovery to characterize drug-target binding and elucidate structure-activity relationships [178]. Recent advancements in understanding MGD highlight SPR’s utility, including in exploring the phytohormone auxin as an MGD for TIR1 and AFB protein [179].

AlphaLISA is a bead-based proximity assay used in high-throughput screening due to its homogeneous format, high sensitivity, and ability to detect various interactions [169,180,181,182,183]. It functions by bringing donor and acceptor beads into proximity upon binding, leading to a luminescence signal. In TPD, AlphaLISA is essential for measuring ternary complex formation mediated by MGDs. While AlphaLISA and TR-FRET are commonly applied, a comparative study revealed that AlphaLISA was more susceptible to chemotype-dependent interference [184].

**Figure 7:**
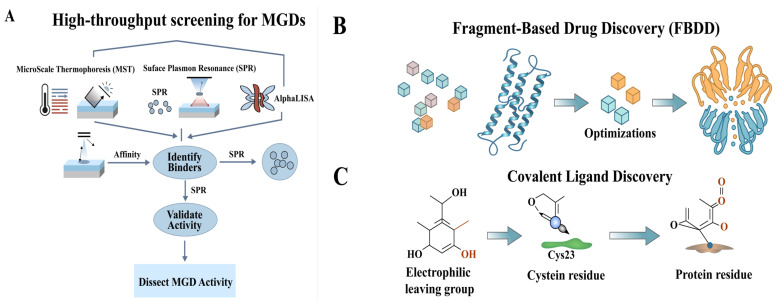
Biophysical and Biochemical Approaches for identifying MGD targets and E3 ligases. (**A**) High-throughput screening for MGDs. Biophysical and biochemical methods such as MicroScale Thermophoresis (MST), Surface Plasmon Resonance (SPR), and AlphaLISA assays are employed to measure binding affinity and activity. These approaches enable the identification of binders, validation of functional activity, and systematic dissection of MGD activity. (**B**) Ligand optimization strategy. Small-molecule binders identified from screening are optimized through iterative cycles of structural refinement, leading to improved binding affinity and specificity toward protein targets. (**C**) Covalent ligand discovery workflow. Electrophilic leaving groups are designed to react with nucleophilic cysteine residues (e.g., Cys23) on target proteins, forming covalent bonds with amino acid side chains and stabilizing protein-ligand complexes for functional degradation activity. The pictures were drawn using Illustrator. Note: MGD, Molecular glue degrader; MST, MicroScale Thermophoresis; SPR, Surface Plasmon Resonance.

Fragment-based drug discovery (FBDD) identifies small, low-molecular-weight fragments that bind weakly but specifically to target proteins, including E3 ubiquitin ligases. These fragments can then be optimized into functional MGDs [185,186,187,188,189]. FBDD is particularly promising for MGD development as it excels at identifying weak interactions within shallow or cryptic pockets, which are crucial for mediating induced PPI [23,51]. Its synergy with structural biology techniques, such as X-ray crystallography and NMR spectroscopy, and computational chemistry tools provides atomic-level insights and optimizes lead compounds, central to MGD activity [185,190] (Fig. 7).

Covalent ligand discovery focuses on identifying electrophilic compounds that covalently modify reactive residues (e.g., cysteines) on E3 ligases, creating new opportunities for proximity-induced protein degradation [38,83,87,191,192]. These ligands form strong, often irreversible bonds, providing increased potency, a prolonged duration of action, and enhanced selectivity [38,193,194,195]. This strategy expands the range of targetable E3 ligases [28,195,196]. Fragment-based screening with reactive groups is a common method [197,198]. An example is the structure-based design targeting the SH2 domain of SOCS2, leading to the covalent inhibitor MN551 [199]. Covalent ligands can also be incorporated into PROTACs [191,196]. Activity-based protein profiling identifies cysteine-reactive small molecules that interact with E3 ubiquitin ligases like RNF4, displaying potential for broadening E3 ligase recruiters for TPD applications [200].

### 5.5 Cell-Based Assays and Functional Profiling

Live-cell E3 ligase activity assays using ubiquitin-binding domain (UBD) reporters offer a powerful tool for dynamic, real-time monitoring of polyubiquitin chain formation in an E3-dependent way, making them useful for screening E3 ligase inhibitors or activators [201,202,203]. These UBD reporters bind specifically to polyubiquitin chains, allowing the direct visualization and tracking of ubiquitin chain formation kinetics and dynamics within living cells [201,202]. The assays are E3 ligase-dependent and responsive to high-throughput screening for the rapid identification of compounds that modulate E3 ligase activity [204].

Scalable assays have been developed to identify compounds that alter the interactome of a specific E3 ligase by tracing its abundance after pharmacologically induced autodegradation and address the challenge of co-opting only a small fraction of E3 ligases for degrader development; this was validated for CRBN [77]. Cell-based target engagement assays evaluate the binding affinity of E3 ligase ligands in living cells, overcoming issues like poor cell permeability encountered *in vitro* [205]. Newer technologies, including NanoLuciferase- and HaloTag-based screenings, enable the determination of kinetics and stability of small-molecule-induced ternary complex formation with live cells [206].

Covalent functionalization and electroporation (COFFEE) are a novel method that involves the covalent labeling of recombinant E3 ligases via maleimide-thiol chemistry, followed by their introduction into cells via electroporation [28,36]. This technique allows the functional analysis of engineered or previously uncharacterized E3 ligases directly within a cellular context [36,37], bypassing the conventional requirement for identifying specific binders. Understanding the roles of E3 ligases in cellular regulation and developing TPD strategies is highly valuable, as demonstrated by the application of this method to various E3 ligases [207]. Furthermore, COFFEE aligns with broader efforts in TPD where covalent recruitment strategies are increasingly utilized. For instance, the discovery of a cysteine-reactive covalent recruiter (EN884) against the SKP1 adapter protein of the SCF complex illustrates that core components of Cullin-RING E3 ubiquitin ligase complexes can be exploited for PROTAC and MGDs applications [208]. These advancements collectively emphasize the potential of utilizing both functionalized E3 ligases and covalent recruiters to target diverse target proteins, thereby expanding the druggable proteosome.

### 5.6 Computational and In Silico Approaches

Computer-aided drug design and artificial intelligence (AI)-driven drug discovery are central to modern MGD research, as they allow the structural exploration of ternary complexes that historically lacked crystallographic resolution. Accurate protein structures predicted by AlphaFold [209,210,211] and RoseTTAFold [212], along with experimental approaches like X-ray crystallography and cryo-electron microscopy (cryo-EM) [17], offer the structural framework required to model degrader-target-E3 ligase assemblies. Using these structures, computational methods, such as molecular docking, molecular dynamics simulations, and free-energy calculations (MM/PBSA, FEP), are used to position degrader molecules within PPI, resolve steric clashes, assess conformational flexibility, and estimate binding affinities [213]. These approaches help identify key interface residues and predict geometries that favor ubiquitin transfer, thus guiding rational degrader design [214].

AI and machine learning (ML) extend these capabilities by analyzing large degradomics and ligandability datasets to predict novel E3-substrate interactions and prioritize compound candidates. Tools like MetaDegron [215] and UbiBrowser [216] allow proteome-wide predictions of degron motifs and ligase-substrate compatibilities, while DL models trained on structural and chemical features predict degrader binding affinity, specificity, and off-target interactions [217,218,219]. Notably, ML models trained on cellular degradation data bridge the gap between initial ternary binding events and functional outcomes by predicting whether binding will translate into productive ubiquitination and target degradation [220,221]. Additionally, AI platforms assess physicochemical and pharmacokinetic properties to filter unsuitable compounds and accelerate lead optimization [222,223].

**Figure 8:**
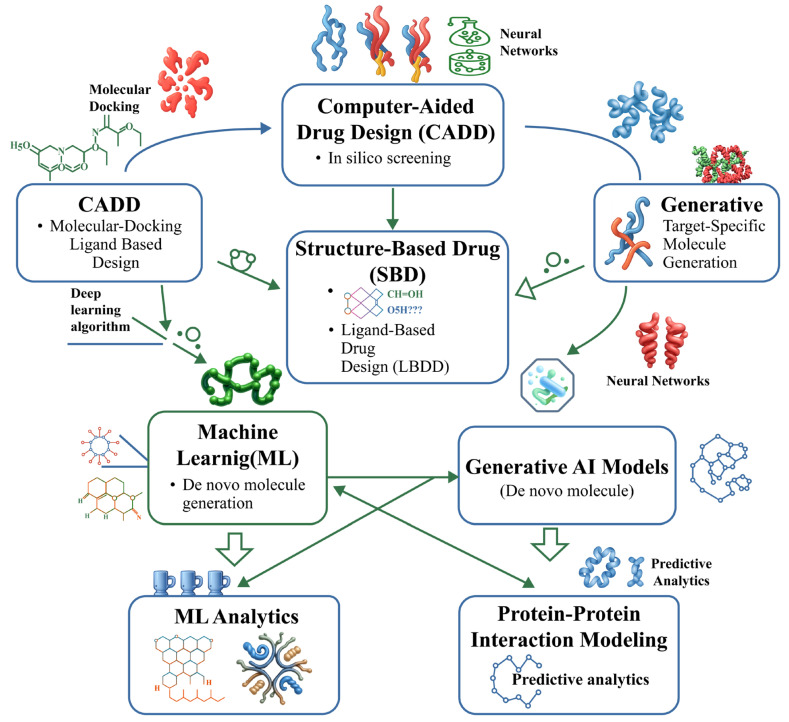
AI-driven drug discovery pipeline for MGDs. This schematic illustrates the integration of computational and artificial intelligence (AI) methodologies to accelerate MGD development. Structure-based drug design (SBDD) serves as the central framework, utilizing three-dimensional protein structures to guide rational ligand optimization. Computer-aided drug design (CADD), encompassing molecular docking and ligand-based design, supports the identification and refinement of candidate molecules. Machine learning (ML) enables large-scale data analysis to predict molecular properties, prioritize drug-like candidates, and inform decision-making. Generative AI models further expand accessible chemical space by designing novel molecules *de novo*. Protein-protein interaction (PPI) modeling predicts and optimizes interactions between target proteins and E3 ligases, a critical step for targeted protein degradation (TPD). Arrows indicate the flow of information between methodologies, highlighting their synergistic integration to accelerate discovery. The pictures were drawn using Illustrator. Note: MGDs, molecular glue degraders; AI, artificial intelligence; SBDD, Structure-based drug design; CADD, Computer-aided drug design; ML, Machine learning; TPD, targeted protein degradation.

Beyond simple prediction, generative AI models represent a paradigm shift by enabling the *de novo* design of MGD. Graph neural networks and variational autoencoders can generate entirely new chemical scaffolds that are not merely derivatives of existing drugs. These models tailor candidate molecules to precisely match the structural features of E3 ligase binding pockets and target surfaces [224,225]. Furthermore, these models conduct multi-parameter optimization to balance potency, selectivity, solubility, and ADME/Tox characteristics [222,226,227], while AI-powered retrosynthesis planning ensures synthetic feasibility by proposing practical chemical routes [228]. Such generative strategies are particularly powerful for designing MGDs against novel E3 ligases, expanding beyond canonical systems like CRBN and DCAF15 [62,229]. This includes exploring unconventional mechanisms, such as CR8-mediated CDK12/cyclin K-DDB1 recruitment and BI-3802-induced BCL6 polymerization [110,114] (Fig. 8).

Because MGDs ultimately function by modulating PPIs, PPI computational modeling is also indispensable. Protein-protein docking predicts possible binding interfaces between targets and ligases, while hotspot analyses highlight residues that contribute most to binding free energy, and molecular dynamics simulations test the stability and ubiquitin accessibility of ternary complexes [213,227,230,231,232]. These models suggest which E3 ligases are structurally compatible with a given target [14] and support virtual screening pipelines that evaluate thousands of candidate molecules *in silico*, prioritizing those most likely to induce degradation [233,234].

Integrating SBDD, AI/ML-driven prediction, generative design, and PPI modeling offers a comprehensive computational toolkit for MGD discovery. These *in silico* methods collectively allow the rational construction of ternary complexes, the prediction of degradation outcomes, and the identification of new ligase-substrate pairs, while also accelerating lead optimization through early filtering of unfavorable compounds. By reducing experimental burden and development timelines, computational and AI-based pipelines are now indispensable for expanding the therapeutic potential of MGDs.

## 6 Strategies for Discovering Novel Neosubstrates for MGDs

Identifying new neosubstrates is essential for expanding the therapeutic potential of MGDs. This process requires a combination of unbiased discovery techniques, targeted functional screens, structural characterization, and computational prediction to elucidate previously unrecognized protein targets that can be selectively degraded through the engagement of E3 ligases.

### 6.1 Global Proteomics-Based Approaches

Degradation-focused quantitative proteomics tracks protein abundance changes upon MGD treatment to identify substrates that are selectively degraded. Stable isotope labeling through SILAC is a robust and efficient metabolic labeling technique, widely used for its accuracy in quantifying protein abundance and determining protein half-lives. It precisely identifies and quantifies isotopically labeled proteins and peptides, making it ideal for studying proteostasis and protein turnover [235,236,237]. Its precision is particularly valuable in studies focusing on cellular signaling dynamics [238]. TMT provides high multiplexing capabilities, allowing for simultaneous, time-resolved analyses of protein degradation and synthesis kinetics, even when combined with SILAC in hyperplexing methodologies [239]. While TMT enables the analysis of more samples concurrently, some studies note that it can have lower coverage than LFQ [238], which provides accurate and robust proteome-wide quantification, especially with methods like MaxLFQ. LFQ is particularly useful for detecting even subtle changes in protein levels across thousands of proteins, making it valuable for understanding cellular proteome remodeling [238,240].

Beyond confirming degradation, quantitative proteomics is crucial in the discovery and mechanistic understanding of MGDs. By profiling protein changes, researchers can pinpoint the specific targets of novel MGDs and unravel their exact mechanisms of action. For instance, chemoproteomic approaches combined with quantitative profiling have been central in identifying new covalent MGDs and their targets, including the oncogenic transcription factor NFKB1 [111]. Similarly, quantitative proteomics has been essential in confirming the degradation activity of MGDs toward specific targets, like cyclin K [110,225], and in elucidating the mechanisms of novel CRBN modulators, including their selective degradation of proteins like GSPT2 [18]. These quantitative methods are also fundamental for evaluating the potency and depth of protein degradation during the lead optimization phase of MGD development [149,241].

Ubiquitome profiling, utilizing ubiquitin-affinity purification coupled with MS, identifies proteins modified by ubiquitin. This method enables researchers to pinpoint potential substrates of ubiquitin ligases, particularly in response to stimuli such as MGDs. By enriching for ubiquitin-modified peptides and analyzing them via MS, researchers can identify specific proteins that undergo ubiquitination. This approach provides a comprehensive map of the ubiquitinated proteome, allowing for a deeper understanding of the biological processes and signaling pathways in which these target proteins are involved [242,243].

The BioE3 method exemplifies this strategy by using E3 ligase fusions, such as CRBN, to detect both existing and novel substrates. Specifically, this approach identifies neosubstrates like CSDE1 upon treatment with MGDs such as pomalidomide. This technique effectively confirms a major rearrangement of the endogenous ubiquitination landscape, providing direct evidence of how MGDs reprogram cellular machinery. By monitoring these shifts, researchers can map the broader impact of induced proximity on the ubiquitin-proteasome system [244]. Polyubiquitin enrichment (via TUBEs) was combined with LC-MS/MS to monitor how small molecules like MGDs alter cellular ubiquitination. This approach is crucial because it identifies specific ubiquitinated proteins and can pinpoint the exact lysine residues to which ubiquitin is attached, as indicated by the detection of non-degradative ubiquitination on UBE3A. It enables the precise characterization of compound-induced ubiquitin-mediated bioactivity [245,246].

Crosslinking mass spectrometry (XL-MS) is a robust technique used to identify direct or near-proximity interactions between proteins, especially transient complexes like those formed by E3 ligases and their substrates [247,248,249,250]. By using crosslinking agents to stabilize these interactions, XL-MS allows researchers to capture and analyze these normally short-lived complexes, providing valuable insights into PPIs and protein structures. XL-MS can pinpoint these newly formed interactions; for example, if an MGD brings a protein (protein A) not typically recognized by an E3 ligase (ligase X) into proximity, XL-MS can identify the cross-links between them, demonstrating that protein A has become a neosubstrate for ligase X.

**Figure 9:**
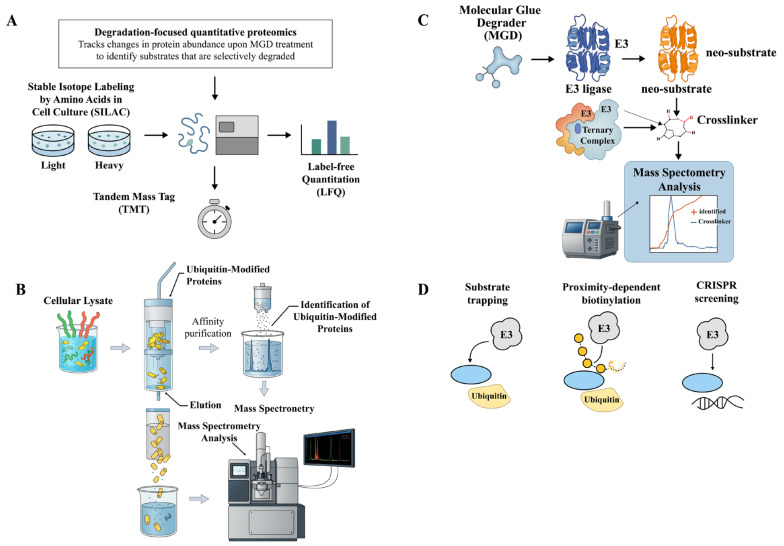
Global Proteomics-Based Approaches for Discovering Novel Neo-Substrates for MGDs. (**A**) Schematic illustration of degradation-focused quantitative proteomics. Degradation-focused quantitative proteomics tracks protein abundance changes following MGD treatment to identify selectively degraded substrates. Stable Isotope Labeling by Amino Acids in Cell Culture (SILAC) offers high accuracy for quantifying protein abundance and turnover, while Tandem Mass Tag (TMT) provides multiplexing capacity for simultaneous, time-resolved proteome analyses. Label-free quantification (LFQ), particularly MaxLFQ, enables robust proteome-wide profiling of subtle abundance changes. (**B**) Schematic illustration of ubiquitome profiling. Ubiquitome profiling employs ubiquitin-affinity enrichment and MS to identify ubiquitinated proteins and map ubiquitination sites in response to MGDs. Techniques such as TUBEs-based polyubiquitin enrichment and BioE3 profiling enable identification of both degradative and non-degradative ubiquitination events, revealing MGD-driven rewiring of the ubiquitin landscape. (**C**) Schematic illustration of crosslinking mass spectrometry (XL-MS). XL-MS stabilizes transient ternary complexes between E3 ligases, MGDs, and substrates, allowing direct detection of novel interactions and identification of previously unrecognized neo-substrates brought into proximity by MGDs. (**D**) Schematic illustration of reverse proteomics strategies. Reverse proteomics strategies start from a candidate protein and identify the responsible E3 ligase(s). Approaches include substrate trapping, proximity-dependent biotinylation, and CRISPR-based functional genomics screens, enabling the discovery of weak or transient E3-substrate interactions and broadening the understanding of ubiquitin signaling networks. The pictures were drawn using Illustrator. Note: SILAC, Stable Isotope Labeling by Amino Acids in Cell Culture; TMT, Tandem Mass Tag, LFQ, Label-free quantification; XL-MS, crosslinking mass spectrometry; MGD; molecular glue degraders; CRISPR, clustered regularly interspaced short palindromic repeats.

Reverse proteomics begins with a known or candidate protein and aims to identify the specific E3 ubiquitin ligase(s) responsible for attaching ubiquitin to it. Analytical methods such as substrate trapping [251,252], proximity-dependent biotinylation [253,254,255], and CRISPR-Cas9 screening [89] allow for the identification of weak and transient interactions between E3 ligases and their target proteins. These techniques are particularly instrumental in uncovering the specific enzymatic machinery that regulates the stability and degradation of a given protein within the ubiquitin-proteasome system (Fig. 9).

### 6.2 Targeted Functional Screens

Targeted functional screens are powerful tools for identifying proteins susceptible to MGD-induced degradation and understanding the regulatory mechanisms involved. CRISPR activation (CRISPRa) and CRISPR inhibition (CRISPRi) libraries, at the forefront of these genetic screens, are used to assess how susceptible proteins are to MGD-induced degradation by precisely controlling their expression levels [256]. These methods use modified Cas9 enzymes (dCas9). CRISPRa uses activating domains to enhance gene expression, while CRISPRi uses repressing domains to suppress it, all without altering the underlying DNA sequence [256]. The process involves introducing a library of CRISPR guide RNAs (gRNAs) into cells. Each gRNA is designed to target a specific gene, allowing for the systematic modulation of thousands of genes in parallel [257,258]. In the context of MGD research, cells are treated with MGD, and the impact of these CRISPR-mediated changes in gene expression on protein levels is measured [256].

If a protein is typically degraded by MGD, lowering its gene expression with CRISPRi would likely reduce its levels. In contrast, if a protein is not affected by the MGD, its levels should remain relatively stable regardless of whether CRISPRa or CRISPRi is employed [256]. These screening strategies offer crucial insights by facilitating the identification of several key components. First, they help pinpoint MGD substrates, which are the specific target proteins directly recognized and degraded by the MGD [256]. Second, these screens reveal regulatory factors, encompassing proteins that influence the kinetics or efficiency of the degradation process itself [256,259]. Finally, they highlight potential therapeutic targets, as identifying the cellular components critical for MGD function can reveal entirely new avenues for therapeutic intervention [256].

**Figure 10:**
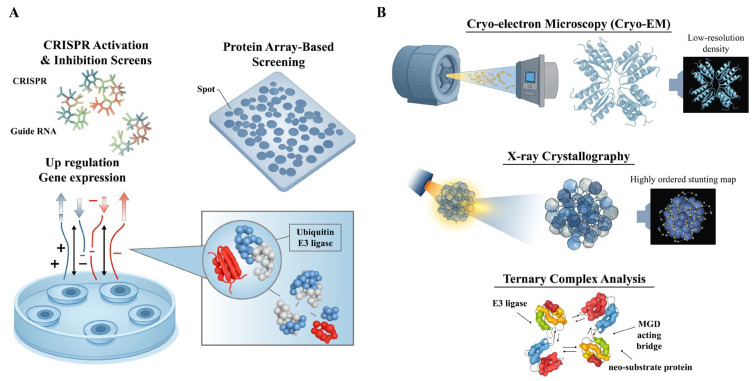
Integrated Approaches for Discovering Novel Neo-Substrates for MGDs. (**A**) Targeted functional screening strategies. Targeted functional screening strategies enable systematic identification of proteins susceptible to MGD-induced degradation. CRISPR activation (CRISPRa) and inhibition (CRISPRi) libraries allow precise modulation of gene expression to evaluate protein stability, while protein array-based screening provides a high-throughput platform to assess E3 ligase-substrate interactions *in vitro*. These approaches facilitate functional validation of potential MGD-responsive targets. (**B**) Schematic illustration of complementary structural biology methods. Complementary structural biology methods provide mechanistic insights into MGD action. Cryo-electron microscopy (Cryo-EM) and X-ray crystallography reveal structural details of E3 ligases, substrates, and MGDs at different resolutions, while ternary complex analysis captures the architecture of MGD-induced E3-substrate assemblies. Together, these approaches combine functional discovery with structural elucidation, enabling a comprehensive understanding of MGD-mediated targeted protein degradation. The pictures were drawn using Illustrator. Note: MGD, Molecular glue degrader; CRISPR, clustered regularly interspaced short palindromic repeats; Cryo-EM, Cryo-electron microscopy.

Protein array-based screening is a high-throughput method that allows researchers to test thousands of purified proteins for MGD-induced PPIs with a known E3 ligase, directly facilitating substrate discovery [260,261]. This method uses protein arrays, which are solid surfaces, typically glass slides, displaying a high density of immobilized proteins [260,261]. By simultaneously testing a large number of proteins against a specific E3 ligase, researchers can rapidly identify potential binding partners [260,262]. The exact definition of MGDs may vary depending on the research context; however, it generally signifies a specific cellular process or pathway in which E3 ligases are fundamentally involved. The ability to directly observe interactions between E3 ligases and their potential substrates on the array allows a faster identification of these critical protein partners [261,262,263]. Identifying E3 ligase substrates is crucial for understanding various cellular processes and disease mechanisms, as well as for potentially developing new therapeutic targets [202,263] (Fig. 10A).

### 6.3 Structural Biology

Structural biology strategies provide atomic-level insights into the intricate interactions within MGD-induced complexes. Cryo-EM and X-ray crystallography are powerful methods that provide high-resolution visualization of ternary complexes formed by E3 ligases, MGDs, and neosubstrates [264,265,266,267,268]. These structural insights clarify the specific interface responsible for substrate recruitment and guide the rational design of new MGDs, as well as the selection of appropriate neosubstrates for TPD [269,270]. These two techniques are complementary: cryo-EM is excellent for visualizing large, flexible complexes and conformational changes induced by MGDs, while X-ray crystallography provides atomic details of smaller, well-ordered structures and high-resolution structures of glue-protein complexes [264,266,271,272].

Structural analysis of these ternary complexes reveals the precise interface where the MGD, E3 ligase, and neosubstrate interact. Visualizing this interface allows researchers to identify key residues or structural features vital for substrate binding and recognition [19,273,274]. The knowledge gained from these analyses can then be used for rational MGD design and substrate selection. Specifically, if a particular region of the neosubstrate is determined to be critical for binding, MGDs can be strategically designed to target that precise area. Furthermore, by understanding the binding preferences of the E3 ligase, researchers can identify additional neosubstrates that are more likely to be targeted for degradation [19,273] This structural insight transitions MGD development from empirical screening to a more predictable, SBDD paradigm (Fig. 10B).

Recent advancements in these fields have revealed how MGDs engage the E3 ligase and target protein for degradation at an atomic level [272]. For example, the CRBN E3 ligase’s recognition of the G-motif in target proteins is essential for how MGDs facilitate target degradation, effectively reprogramming the ligase’s substrate specificity to create a neosubstrate or degron [229]. This knowledge is widely applicable in drug discovery and development, where MGDs are emerging as a promising new class of therapeutics [264]. The two techniques are indispensable for understanding how MGDs bind to their target proteins and E3 ligases, and how this binding leads to conformational changes that trigger protein degradation or other cellular effects [19,273,274].

### 6.4 Computational and In Silico Approaches

#### 6.4.1 Molecular Docking and MD Simulations in MGD Discovery

Computational and *in silico* approaches play an increasingly vital role in predicting, modeling, and optimizing MGDs and TPD strategies. Molecular docking and molecular dynamics (MD) simulations are computational tools that model how MGDs induce or stabilize interactions between an E3 ligase and candidate substrates, thus predicting their binding compatibility [275,276]. These *in silico* methods overcome the limitations of rigid sampling in docking analysis by offering valuable insights into the functional mechanisms of biomolecules [276]. Specifically, molecular docking can predict the binding affinity and interactions between proteins and ligands, while MD simulations offer dynamic insights into the structural and functional changes that occur upon binding [276]. For example, these techniques have been used to model the interaction between the FAK inhibitor VS-4718 and its target, revealing key hydrogen bonds and hydrophobic interactions crucial for binding [277].

#### 6.4.2 Rational Design through PPI Modeling

In the context of MGDs, which are monovalent small molecules that either stabilize native PPIs or induce neomorphic interactions [19], computational methods are becoming essential for their discovery. Molecular docking and MD simulations can help identify potential hotspots on target proteins and E3 ligases where MGDs might effectively mediate and enhance PPIs [19]. For instance, these methods can be used to elaborate derivatives of known ligands, contributing additional hotspots to a PPI interface and increasing affinity for new protein interactors [278]. Furthermore, computational approaches can explore allosteric sites where MGDs might bind to induce or modify a PPI interface by stabilizing a non-native protein conformation, a process that can leverage existing databases of structural ensembles [279,280].

#### 6.4.3 AI and Machine Learning for Predicting Protein Degradability

While serendipity has been significant in MGD discovery, advancements in AI and ML are crucial for overcoming current challenges in TPD [279]. ML models trained on degradomics data are being developed to predict the degradability of proteins based on their sequence, structure, or interactome features. A prominent example is the Model-free Analysis of Protein Degradability (MAPD), developed to predict the degradability of proteins, particularly kinases, from their intrinsic features [281]. MAPD exhibited accurate performance, revealing that E2-accessible ubiquitination sites are particularly associated with kinase degradability. These predictions have been extended to the entire proteome, identifying thousands of disease-causing proteins, including cancer-related genes, as potential TPD drug targets [281].

#### 6.4.4 Specialized Predictive Models: PrePROTAC and DeepPROTACs

Similarly, PrePROTAC, an interpretable ML model based on a transformer-based protein sequence descriptor, was developed to predict genome-wide PROTAC-induced targets degradable by CRBN [282]. PrePROTAC has already identified over 600 new proteins potentially degradable by CRBN, including targets for Alzheimer’s disease. To address challenges in rational design, the DeepPROTACs deep neural network model predicts the degradation capacity of a proposed molecule based on the structures of the target protein and E3 ligase [283]. This model, which uses graph convolutional networks for feature extraction, achieved an average prediction accuracy of nearly 78%, thereby reducing the time and cost associated with drug discovery by enabling virtual screening before synthesis [283,284].

#### 6.4.5 Deep Learning Frameworks for Degron Mapping and Neosubstrate Identification

**Figure 11:**
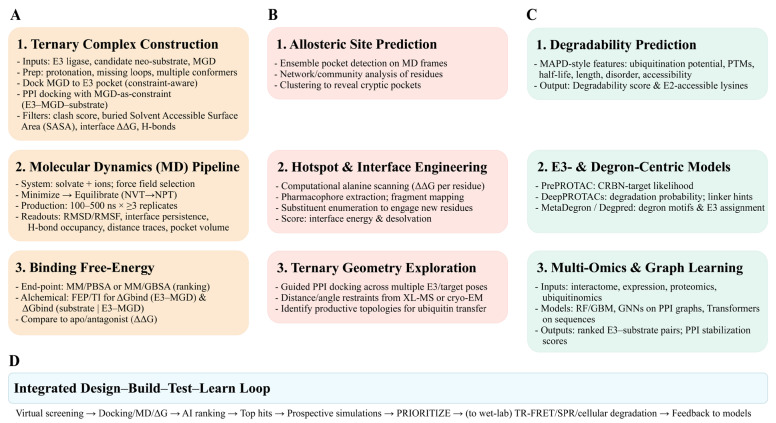
Computational & *in silico* methods for MGDs & TPD-Method-Centric workflow. The workflow summarizes three methodological domains: (**A**) Structure-based modeling. Structure-based modeling, including ternary complex construction, molecular dynamics simulations, and binding free-energy estimation; (**B**) Pocket and hotspot discovery. Pocket and hotspot discovery, encompasses allosteric site prediction, interface engineering, and ternary geometry exploration; and (**C**) Data-driven prediction. Data-driven prediction uses AI/ML, incorporating degradability scoring, E3- and degron-centric models, and multi-omics with graph learning. (**D**) These approaches are integrated through an iterative Design → Build→ Test → Learn loop to guide virtual screening, target prioritization, and feedback to model refinement. The pictures were drawn using Illustrator. Note: MD, Molecular Dynamics; PPI, protein-protein interaction.

The field is also advancing in predicting degrons-the short linear amino acid motifs on target proteins recognized by E3 ligases. MetaDegron, a novel DL framework, predicts E3 ligase-targeted degrons by integrating protein language models and comprehensive featurization strategies [215]. Another DL model, Degpred, directly predicts general degrons from protein sequences, expanding the known degron landscape and building a regulatory network of protein degradation [84]. Identifying neosubstrates is crucial for expanding the therapeutic potential of MGDs, requiring a combination of unbiased discovery techniques, structural characterization, and computational prediction to elucidate previously unrecognized protein targets that can be selectively degraded through the engagement of E3 ligases (Fig. 11).

## 7 Implications and Challenges in the Clinical Development of MGD Anticancer Drugs

MGDs have emerged as an innovative therapeutic modality in targeted cancer therapy by reprogramming the ubiquitin-proteasome system to selectively degrade previously “undruggable” oncoproteins. Their ability to induce proximity between an E3 ligase and a target protein offers a fundamentally new pharmacological mechanism compared with conventional inhibitors, with the potential to yield more durable and context-specific therapeutic effects. Several MGDs are currently being clinically evaluated for hematologic and solid malignancies, highlighting their translational promise [18,263,285].

However, despite this potential, the clinical translation of MGDs faces multiple unresolved challenges: First, structural and thermodynamic constraints often make achieving precise substrate selectivity difficult. Second, the context-dependent variability of E3 ligase expression and activity can limit consistent therapeutic efficacy across different tissues. Third, there remains a limited structural understanding of ternary complex formation for many emerging targets. Finally, the emergence of resistance mechanisms, which mirror or even exceed those observed with kinase inhibitors, poses a significant hurdle. Addressing these limitations through interdisciplinary research will be essential for realizing the full therapeutic potential of MGDs.

### 7.1 Specificity and Off-Target Effects in Cancer Therapy

The therapeutic efficacy and safety of MGDs critically depend on their ability to mediate precise E3 ligase-substrate interactions. However, achieving this specificity remains significantly challenging. Many E3 ligases exhibit broad or incompletely characterized substrate repertoires, making it difficult to predict or prevent off-target degradation [285]. Structurally, this challenge arises from the shallow, solvent-exposed, and often flexible binding interfaces characteristic of E3 ligases, such as CRBN and DCAF15. These features permit promiscuous molecular recognition and small energy barriers between productive and nonproductive ternary complexes [286].

From a thermodynamic perspective, the cooperativity driving ternary complex formation is frequently marginal, with ΔΔG values small enough that subtle changes in MGD conformation or cellular conditions can redirect binding toward unintended substrates. Moreover, the dynamic nature of protein surfaces and the entropic contributions of weak hydrophobic contacts complicate the prediction of selectivity. These molecular properties explain why off-target effects persist even in rationally designed MGDs [286].

Recent efforts to address these limitations include high-throughput mapping of E3-substrate networks, structure-based design to enhance binding cooperativity, and computational modeling to predict alternative interaction geometries [35,51]. However, these approaches remain hindered by incomplete structural data and the lack of a comprehensive E3-substrate interaction atlas [35]. Thus, despite notable progress, achieving true selectivity remains a central translational obstacle for MGD therapeutics.

### 7.2 Cellular Context and E3 Ligase Expression

E3 ligase expression and activity are highly variable across different tissues, developmental stages, and disease states, profoundly influencing both the efficacy and safety of MGDs. For instance, a ligase that is abundant in hematologic malignancies may be inactive or absent in solid tumors [38,287]. This heterogeneity extends beyond expression levels to include subcellular localization, post-translational modifications, and competition with endogenous substrates [263,288]. Consequently, MGDs optimized in one cellular context may not function effectively in another.

While this variability poses a significant translational challenge, it also offers unique therapeutic opportunities for achieving tissue-specific degradation and reducing systemic toxicity [289]. Future studies integrating single-cell transcriptomics, proteomics, and spatial proteome mapping will be essential to precisely match E3 ligase availability with appropriate disease contexts [16,288,289,290,291]. By leveraging these insights, we can design MGDs that utilize ligases exclusively expressed in diseased tissues, thereby enhancing the therapeutic index and minimizing off-target effects in healthy cells.

### 7.3 Mechanistic Elucidation and Structural Validation

A persistent limitation in MGD development is the incomplete mechanistic understanding of ternary complex formation. Biochemical assays can reveal degradation but not the structural determinants that govern selectivity or cooperativity [292]. High-resolution methods, including X-ray crystallography and cryo-EM, remain indispensable to define how MGDs reorganize E3 ligase-substrate interfaces at the atomic level [265,293,294,295,296].

Recent structural studies have revealed that minimal chemical modifications-such as altering hydrogen bond donors or aromatic substituents-can significantly impact the stability and productivity of ternary complexes [18]. Without such precise structural information, many promising MGDs may fail to achieve efficient ubiquitination of the target protein. This underscores the critical necessity for parallel structural and biochemical validation during the preclinical development phase.

### 7.4 From CRBN to VHL: Broadening the Druggable E3 Ligases Space for MGDs

Despite the vast potential of E3 ligases, their intrinsic structural properties-namely their large, featureless, and flexible protein surfaces-render most of them poorly druggable by conventional small molecules, traditionally limiting the usable repertoire to a handful of well-characterized ligases such as CRBN and MDM2 [28,29,289]. To circumvent these constraints, innovative approaches including covalent ligand discovery, FBDD and the use of engineered or viral E3 ligases with restricted tissue expression have been explored; however, each of these carries distinct translational challenges in terms of selectivity, reversibility, and clinical safety [23,51,185,297]. Consequently, improving the “ligase druggability landscape” remains a critical priority for the development of next-generation MGDs, implying the utilization of a wider array of E3 ligases for clinical application. While the limitations of CRBN-based MGDs are well established, the E3 ligase platform is rapidly expanding with promising alternatives, notably other DCAF ligases, among which the VHL ligase has rapidly emerged as a critical and actionable platform. The discovery of a small molecule that binds the HIF1-α-binding pocket on VHL and acts as an MGD by recruiting CDO1 as a neosubstrate into the VHL E3 ligase complex, clearly demonstrates that VHL has moved beyond a merely potential target [65]. Furthermore, an independent report identifying dGEM, a novel MGD that recruits the VHL E3 ligase to the neosubstrate GEMIN [298], further reinforces the reproducibility and clinical relevance of the VHL MGD approach. These two distinct, well-characterized VHL MGD examples suggest that claims regarding VHL’s addressability via MGDs are accurate at this juncture, which carries the significant implication of expanding the range of clinically targetable ligases beyond CRBN to novel E3 platforms.

### 7.5 Computational Model Development

Computational modeling has begun to inform the rational design of MGDs by predicting degron motifs, E3-substrate compatibilities, and binding cooperativity [14,213,227,299]. However, these models remain largely descriptive due to several critical limitations, such as incomplete training datasets, a lack of diverse structural templates, and the highly dynamic nature of cellular protein networks [35,39,84].

To improve predictive accuracy, future computational frameworks must integrate context-specific parameters. First, these models should incorporate cell type-specific E3 ligase expression profiles. Second, they must account for the impact of post-translational modifications on protein interactions. Finally, the models need to reflect the conformational flexibility inherent in ternary complex formation [300]. Until such integrative models are fully realized, computational predictions must be empirically validated through quantitative proteomics and structural analysis to ensure translational reliability. This synergy between *in silico* prediction and experimental validation is essential for the robust development of next-MGDs.

### 7.6 Overcoming Drug Resistance and Expanding Therapeutic Options

Resistance to MGDs has already been observed and may arise through several mechanisms, including mutations in the E3 ligase (e.g., CRBN) or the neosubstrate, the loss of essential cofactors such as DDB1 or CUL4, or the compensatory activation of redundant degradation pathways [21,58,80,167,301]. Comparative analysis of strategies to overcome such resistance reveals distinct advantages and trade-offs. First, ligase switching-the recruitment of alternative ligases-can bypass mutation-induced resistance; however, this approach requires compatible degron motifs and rigorous structural validation [29,302]. Second, ligand re-engineering aimed at improving binding cooperativity offers a modular approach to restore efficacy, though it may inadvertently broaden the substrate profiles. Third, combination therapies involving chemotherapy, targeted agents, or immunotherapy can mitigate adaptive resistance, yet they carry the risk of cumulative toxicity [121]. Finally, systems-level approaches, such as CRISPR-based functional genomics, can systematically identify resistance nodes and suggest potential compensatory pathways [89].

Critically, these strategies differ mechanistically and in terms of translational feasibility and safety. This diversity highlights the necessity for context-dependent optimization tailored to specific disease environments, rather than relying on one-size-fits-all solutions.

### 7.7 Expanding Ligase Repertoires and Discovering Novel Neosubstrates

Despite over 600 putative human E3 ligases, most MGD research remains confined to a few canonical examples [36,67,303]. Expanding the ligase repertoire is thus essential to broaden therapeutic reach, overcome resistance, and achieve tissue- or pathway-specific control. Recent advances, including BioE3, COFFEE, and degron mapping, have enhanced the detection of transient ligase-substrate interactions [28,36,255,304]. Integrating these tools with multiomics datasets, including proteomics and single-cell transcriptomics, promises to accelerate the discovery of new degradable targets.

Moreover, the continuous identification of neosubstrates, such as CK1 α and other CRBN-dependent oncogenic proteins, extends the potential of MGDs beyond hematologic malignancies to solid tumors [16,17,121,289,305]. However, many newly reported targets lack validation of ternary complex formation or degradation kinetics, underscoring the ongoing need for stringent structural and biochemical confirmation.

Overall, while MGDs represent a transformative paradigm in cancer therapy, their successful clinical translation requires a deeper mechanistic understanding of E3-substrate specificity, structural cooperativity, and the evolution of resistance. A critical synthesis of successes and limitations, encompassing thermodynamics, ligand design, and cellular context, will be crucial for guiding the development of the next generation of safe and effective MGD-based anticancer agents.

## 8 Conclusion and Perspectives

MGDs have emerged as a powerful therapeutic paradigm by enabling the selective elimination of oncogenic proteins that were previously considered undruggable. Through neomorphic interactions between E3 ubiquitin ligases and neosubstrates, MGDs harness the ubiquitin-proteasome system to achieve potent and durable protein degradation. Clinical validation with CRBN-based MGDs has transformed the treatment landscape in hematologic malignancies; yet reliance on a small set of ligases limits substrate diversity, tissue selectivity, and long-term disease control.

Early clinical outcomes underscore the direct anticancer potential of MGDs. Thalidomide analogs, such as iberdomide and avadomide, have demonstrated strong efficacy in multiple myeloma and lymphoma. In contrast, arylsulfonamide-based degraders, including indisulam and E7820, have displayed therapeutic activity against the splicing factor RBM39 in acute myeloid leukemia and select solid tumors. Similarly, the CRBN-dependent degrader CC-90009, which targets GSPT1, has resulted in the rapid clearance of leukemic progenitors in AML patients. These results underscore the ability of MGDs to achieve clinically meaningful responses across diverse cancer types, providing a foundation for developing next-generation degraders as precision anticancer drugs.

Future progress in this field will depend on expanding beyond CBRN and VHL to harness the broader repertoire of more than 600 putative human E3 ligases. Identifying ligases characterized by tissue-restricted expression, structural tractability, and high substrate specificity will be crucial for enabling selective and durable therapeutic responses. Advances in diverse methodologies-specifically phenotypic screening, chemical proteomics, ubiquitin-remnant profiling, CRISPR-based functional genomics, and AI-driven structural modeling-are rapidly accelerating the discovery of novel ligase-substrate networks. Simultaneously, understanding and anticipating resistance mechanisms, such as mutations at the ligase-substrate interface observed with CRBN and DCAF15, will be critical to ensure long-term clinical efficacy.

Looking ahead, progress will require close collaboration among chemists, structural biologists, and translational oncologists to successfully transition MGDs from discovery to clinical practice. By expanding the ligase-neosubstrate universe and integrating MGDs into multimodal cancer therapy, this field has the potential to redefine oncology. This evolution will offer customizable degraders tailored to specific tumor biology and patient-specific contexts. Ultimately, MGDs represent both a technological breakthrough and a clear path toward durable, precision-driven cancer treatment.

## Data Availability

Not applicable.

## Abbreviations

AI	Artificial intelligence
CC	Cervical cancer
DL	Deep-learning
DOI	Digital object identifier
FBDD	Fragment-based drug discovery
IMiD	Immunomodulatory drugs
LFQ	Label-free quantification
MAPD	Model-free analysis of protein degradability
MD	Molecular dynamics
MDS	Myelodysplastic syndromes
MGD	Molecular glue degraders
ML	Machine learning
NHL	Non-Hodgkin’s lymphoma
ORR	Overall response rate
POI	Protein of interest
PROTAC	Proteolysis-targeting chimeras
RING	Really interesting new gene
RRMM	Relapsed/refractory multiple myeloma
SILAC	Stable isotope labeling by amino acids in cell culture
SPR	Surface plasmon resonance
TMT	Tandem mass tag
TPD	Targeted protein degradation
TPP	Thermal proteome profiling
UBD	Ubiquitin-binding domain
VHL	Von Hippel-Lindau
XIAP	X-linked inhibitor of apoptosis
